# Global and local evolutionary dynamics of Dengue virus serotypes 1, 3, and 4

**DOI:** 10.1017/S0950268823000924

**Published:** 2023-06-09

**Authors:** Arshi Islam, Farah Deeba, Bansidhar Tarai, Ekta Gupta, Irshad H. Naqvi, Mohd. Abdullah, Ravins Dohare, Anwar Ahmed, Fahad N. Almajhdi, Tajamul Hussain, Shama Parveen

**Affiliations:** 1 Centre for Interdisciplinary Research in Basic Sciences, Jamia Millia Islamia, New Delhi, India; 2 Department of Microbiology and Infection Control, Max Superspeciality Hospital, New Delhi, India; 3 Department of Clinical Virology, Institute of Liver and Biliary Sciences, New Delhi, India; 4 Dr. M.A. Ansari Health Centre, Jamia Millia Islamia, New Delhi, India; 5 Centre of Excellence in Biotechnology Research, College of Science, King Saud University, Riyadh, Saudi Arabia

**Keywords:** Bayesian analysis, Dengue virus, genotype, phylogenetic analysis, serotype

## Abstract

Evolutionary studies on Dengue virus (DENV) in endemic regions are necessary since naturally occurring mutations may lead to genotypic variations or shifts in serotypes, which may lead to future outbreaks. Our study comprehends the evolutionary dynamics of DENV, using phylogenetic, molecular clock, skyline plots, network, selection pressure, and entropy analyses based on partial CprM gene sequences. We have collected 250 samples, 161 in 2017 and 89 in 2018. Details for the 2017 samples were published in our previous article and that of 2018 are presented in this study. Further evolutionary analysis was carried out using 800 sequences, which incorporate the study and global sequences from GenBank: DENV-1 (*n* = 240), DENV-3 (*n* = 374), and DENV-4 (*n* = 186), identified during 1944–2020, 1956–2020, and 1956–2021, respectively. Genotypes V, III, and I were identified as the predominant genotypes of the DENV-1, DENV-3, and DENV-4 serotypes, respectively. The rate of nucleotide substitution was found highest in DENV-3 (7.90 × 10^−4^ s/s/y), followed by DENV-4 (6.23 × 10^−4^ s/s/y) and DENV-1 (5.99 × 10^−4^ s/s/y). The Bayesian skyline plots of the Indian strains revealed dissimilar patterns amongst the population size of the three serotypes. Network analyses showed the presence of different clusters within the prevalent genotypes. The data presented in this study will assist in supplementing the measures for vaccine development against DENV.

## Introduction

Dengue infection in humans is widespread and mainly occurs in tropical and subtropical geographical locations, with an estimated occurrence of 400 million annual infections globally [1]. The disease, caused by the Dengue virus (DENV), ranges from an acute febrile illness known as Dengue fever (DF) to life-threatening Dengue haemorrhagic fever (DHF) or Dengue shock syndrome (DSS) [2]. DENV is classified into the family *Flaviviridae* and genus *Flavivirus.* The approximate length of the positive-sense, single-stranded RNA Dengue virus genome is 10.7 kb, which encodes an open reading frame that translates into three structural (capsid, C; membrane, M; and envelope, E) and seven non-structural proteins (NS1, NS2A, NS2B, NS3, NS4A, NS4B, and NS5) [3].

Various locations in the world have become hyperendemic due to the occurrence of antigenically distinct serotypes of the Dengue virus. Globally, most of the Dengue outbreak cases were reported in the Western Pacific Region after 2010, with cases mostly being identified in China, Malaysia, and Singapore [4]. These frequent life-threatening outbreaks poorly affect a country’s economy and its health system. Apart from the aforementioned Asian countries, the Indian subcontinent has shown a complex epidemiological pattern of Dengue infection in reference to the prevalent strains of the antigenically distinct DENV serotypes, which further impacts the severity of the disease [5]. The existence of the Dengue virus in India was first reported in the year 1946 [6]. Since that breakthrough, many outbreaks with a significant proportion of deaths have been reported from different geographical regions of the country [7–9].

The genotypic variations of the Dengue virus contribute majorly to such outbreaks or epidemics. Hence, for a high-throughput vaccine-designing protocol and, eventually, to combat the severe consequences of the disease, regular molecular surveillance of DENV is much needed so that genotypes can be established and their further dispersal in a country can be avoided. In view of the same, the present study aims to comprehend the diversity of the Dengue virus serotypes by analysing their genotypic relationships, origin, circulation pattern, and global expansion.

In the present study, we have carried out a CprM gene-based phylogenetic and molecular clock analysis of the Dengue virus, including the study and global strains. The study strains of the DENV were identified in 2017 and 2018, in our investigation. Site-specific selection pressures and entropy analyses were also performed to detect the sites that are prone to mutation in this conserved region of the DENV genome. We have also analysed the genotypic relationships of the Dengue virus using median-joining networks of the respective serotypes. Locally, the discrepancies in the Indian population of a particular serotype were also analysed using Bayesian skyline plots.

## Materials and methods

### Sample collection

Patients with Dengue fever–like symptoms were recruited for the present study. The study was approved by Institutional Ethics Committee, Jamia Millia Islamia (JMI), and it was performed in accordance with the World Medical Association Declaration of Helsinki. The blood samples were collected from the Out Patient Department (OPD) of the Dr. M.A. Ansari Health Centre of the university. The clinical proformas and written informed consent (in both Hindi and English languages) of the enrolled patients were also maintained by the clinicians.

### RNA extraction from the serum samples

The process of centrifugation was used for the separation of the serum from the collected blood samples. The samples were centrifuged at a speed of 3,000 rpm for about 10 minutes at 4°C. The total viral RNA was isolated from the 140 μl serum samples using QIAamp Viral RNA Mini Kit (Qiagen, Germany), according to the manufacturer’s instructions. The extracted RNA was stored at −80°C.

### cDNA synthesis

The extracted RNA from the serum samples was used for the synthesis of cDNA. The reaction was carried out using the commercially available High Capacity cDNA Reverse Transcription Kit (Applied Biosystems, Waltham, MA). The reaction conditions to perform cDNA synthesis were already standardised in our laboratory [10].

### Serotyping of Dengue virus

The detection of the Dengue virus was done by external PCR using forward and reverse primer. The serotype-specific PCR (semi-nested) for the conserved CprM region of the Dengue virus was carried out using primers published by Lanciotti et al. [11]. Both external and semi-nested PCR reactions were performed in accordance with the standardised protocol in our laboratory [10].

### Amplification and sequencing

The semi-nested PCR was used for the amplification of the partial sequence of the CprM region of the identified DENV strains. The DNA was further extracted from the agarose gel using the commercially available Plus DNA Clean/ Extraction Kit (GeneMark, Taiwan) in accordance with the instructions provided by the manufacturers. To avoid any uncertainty in obtaining the consensus sequences, the sequencing was done in both forward and reverse directions using outsource services (Applied Biosystems, Waltham, MA). The ambiguities were resolved manually from the nucleotide sequences using the softwares GeneDoc (v2.7) and BioEdit (v.7.2). The obtained consensus sequences were confirmed by the online BLAST tool at NCBI (http://blast.ncbi.nlm.nih.gov/Blast.cgi) prior to the analyses.

### Phylogenetic analysis

The phylogenetic trees for DENV were constructed using sequences published in GenBank. The alignment of the sequences was done in BioEdit (v.7.2) software. The tree was constructed using the maximum likelihood method in MEGA6 (v 6.06) software [12]. The model for the tree construction in MEGA6 was selected by Akaike information criterion (AIC) using jModelTest2 on XSEDE (2.1.6), CIPRES portal [13]. The robustness of the tree was assessed with 1,000 bootstrap replicates. The prototype strains used for the phylogenetic analysis were US/Hawaii/1944 of DENV-1 (Accession number: EU848545), H87 strain of DENV-3 (Accession number: M93130), and H241 strain of DENV-4 (Accession number: KR011349).

### Data selection

Sequences from the same year were down-sampled when they fell within a monophyletic group in the same genotype and same country. Following this downsizing, subsequent root-to-tip analysis was performed on TempEST v1.5.3 to assess the strength of the signal in the resulting dataset. The TempEst requires a ‘nonclock’ phylogenetic tree which utilises branch lengths scaled as genetic distances instead of temporal distances. This tree was obtained by IQ-TREE software (package available on CIPRES portal v2.1.2) using maximum likelihood. The nucleotide substitution model of the resulting dataset was selected on the basis of AIC using jModelTest2.

### Bayesian Markov Chain Monte-Carlo analysis

The analysis was performed using strict and relaxed molecular clock. The relaxed molecular clock includes uncorrelated lognormal and exponential models. Three demographic models were used with each clock. The models were constant size, Bayesian skyline, and exponential growth. At least two independent runs were performed for each combination of the clock and demographic model. The Markov Chain Monte-Carlo (MCMC) chain was run at a length of 60 million (DENV-3 and DENV-4) and 100 million (DENV-1). The best-fit model was chosen by path-sampling and stepping-stone methods. The two tree files and the two log files of the selected best-fit model were combined using LogCombiner 1.10.4 (implemented in BEASTv1.10.4) and Tracer v1.7.1, respectively, with 10% burn-ins removed from each run. The resulting log files were analysed in Tracer to ascertain the convergence of the MCMC chain and to ensure that an effective sample size of (ESS) >200 for all parameters was achieved. The uncertainty in the estimates of the parameters was assessed by 95% HPD interval. The maximum clade credibility tree was generated by Tree Annotator 1.10.4 (implemented in BEAST), and the resulting tree file was visualised in the program FigTree 1.4.4. Support for the node on the tree was ascertained by the Bayesian posterior probability (BPP) values for each node.

### Bayesian skyline plots

The Bayesian skyline plots (BSP) of the Indian DENV strains were also inferred using this BEAST package that enabled a graphical depiction of changing levels of population size through time. The graphical interpretation elucidated the variations in the median estimation of relative hereditary diversity (Ne τ) of DENV with time (Ne and τ denote effective population size and generation time, respectively). The analysis was done using the MCMC approach implemented in the BEAST package (v1.10.4). The skyline plot was generated from the MCMC output using Tracer v1.7.1.

### Median-joining network analysis

The median-joining network was used to visualise genetic relationships in the nucleotide sequences of the CprM region of DENV. The network was constructed using Network 10.2 software. The analysis was performed with the datasets used for the construction of a phylogenetic tree. DnaSP v. 5.10.01 software was used for the preparation of an alignment file for further use in network analysis.

### Selection pressure analysis

The CprM region of DENV was subjected to selection pressure analysis using the online web server Datamonkey (http://www.datamonkey.org). The ratio of non-synonymous to synonymous mutations (dN/dS) was determined using four different analytical methods: single likelihood ancestor counting (SLAC), fixed effects likelihood (FEL), mixed effects model of evolution (MEME), and fast, unconstrained Bayesian approximation (FUBAR). The genomic region selection can be categorised into three types in accordance with the ratio of mean dN/dS: negatively selected when the ratio is less than 1; positively selected when the ratio is greater than 1; and neutral selection when the ratio is equal to 1. In this study, the different parameters (*p*-value and posterior probability) were considered for a site to be positively selected. We have considered those codon positions under positive selection which were selected by at least two different analytical methods.

### Shannon entropy analysis

The amino acid sequences of the CprM region of DENV were subjected to Shannon entropy analysis in BioEdit (v.7.2.) software. A high entropy score at a particular site signifies an increased likelihood of variation at that position. The final data was illustrated as an entropy graph between residue numbers (alignment positions) versus entropy (Hx).

## Results

### Characteristics of the enrolled patients

**Figure 1.**
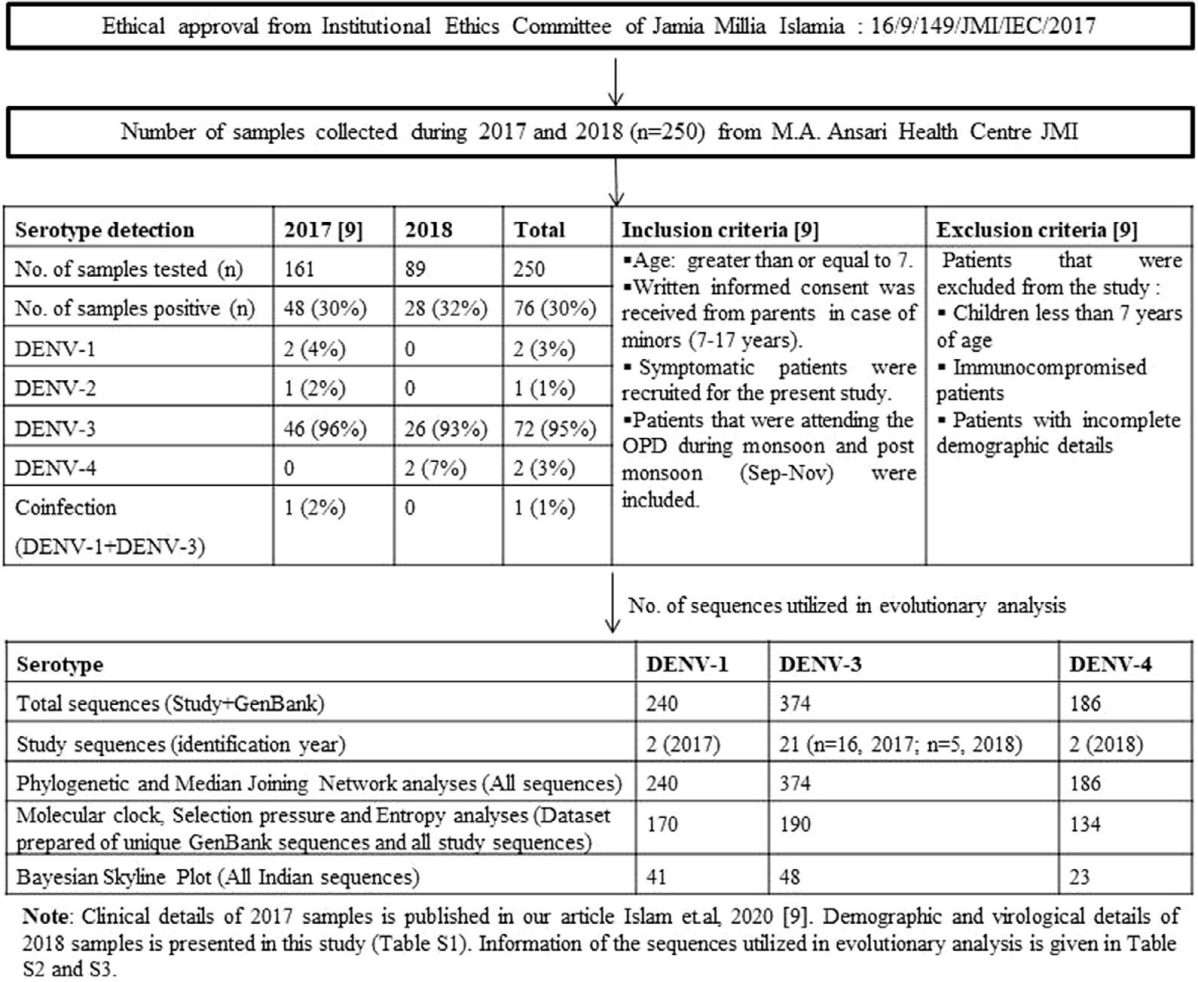
Overview of the study. The figure illustrates the inclusion and exclusion criteria for the sample collection, serotype detection during 2017 and 2018, and the numeric details of the sequences utilised in evolutionary analysis.

Eligible subjects attending the Out Patient Department (OPD) of the Dr. M.A. Ansari Health Centre at the Jamia Millia Islamia, New Delhi, were recruited for the present study. Fever was observed in almost all the patients enrolled in the study. The other symptoms were rashes, headache, vomiting, nausea, retro-orbital pain, and weakness. None of the patients was found infected with critical conditions of Dengue fever (DHF/DSS). The inclusion and exclusion criteria for all the samples collected in the study during 2017 and 2018 (*n* = 250) are given in Figure 1 and are already published in our previous article [9]. The demographic and serotype detection details of the patients enrolled in the study during 2018 are given in Supplementary Table S1. The clinical details for the 2017 samples are published in our previous article [9].

### Serotype detection during 2018

A total of 89 serum samples were tested for DENV during the post-monsoon season of 2018. Twenty-eight samples (32%) were identified as positive for DENV infection. DENV-3 and DENV-4 serotypes were identified in the study with the predominance of DENV-3. Twenty-six samples (93%) were positive for DENV-3 and two samples (7%) were positive for DENV-4 (Figure 1).

### DNA sequencing and data selection

The partial sequences of the CprM region were used for the analysis. Five samples were sequenced for DENV-3 and two for DENV-4 in 2018. The obtained consensus sequences were verified by the BLAST tool at NCBI. We have also utilised our study sequences of DENV-1(*n* = 2) and DENV-3 (*n* = 16) identified in 2017 for the evolutionary analysis. The numeric details of the sequences used in evolutionary analysis are given in Figure 1. The serotypic and genotypic details of the 2017 and 2018 study strains with their accession numbers are given in Supplementary Table S2. Further details and selection of the sequences for evolutionary analyses of DENV-1, DENV-3, and DENV-4 are provided in Supplementary Table S3.

### Phylogenetic analysis of Dengue virus 1

**Figure 2.**
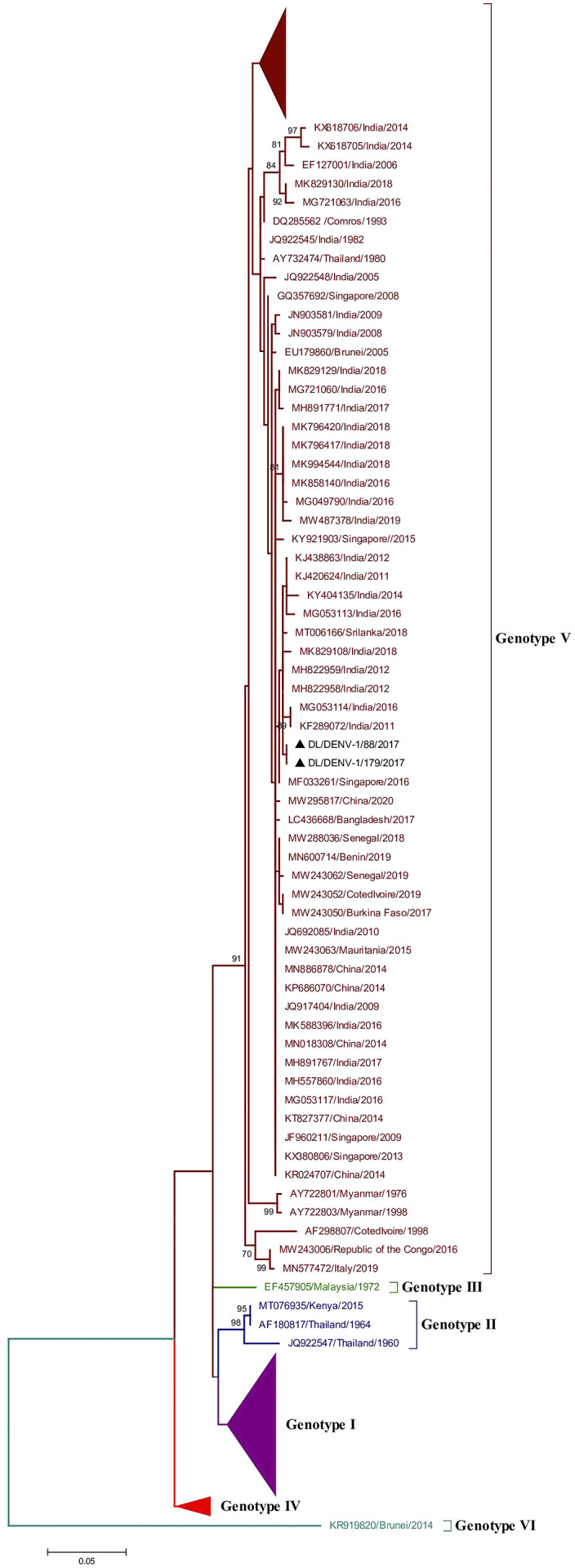
Phylogenetic tree of DENV-1 (*n* = 240). The tree was generated using the maximum likelihood approach using partial CprM gene sequences of DENV-1. The study sequences clustered in genotype V are highlighted in black with the symbol ▲. The arrows are used to provide more clarity to the illustration. The sequences used in the tree are shown by their accession number followed by country and the date of collection.

The phylogenetic tree of DENV-1 was generated using 240 partial CprM gene sequences that also include the two study strains identified in 2017 in our investigation. The length of the aligned region was 429 bp (143 amino acids), which corresponds to 135 to 563 bp of the full genome of the prototype strain. The study sequences got assembled within genotype V along with other Indian strains (Figure 2 and Supplementary Figure S1).

Eight mutations (V26G, L46M, M51I, G70S, L72F, N90S, S93N, and I150L) were identified in the study strains when compared to the full-length amino acid sequence of the prototype strain. These mutations were also reported in earlier studies [6]. The study strains showed a genetic distance of 6.4% and 5.8% at nucleotide and amino acid levels, respectively, in contrast to the prototype strain.

### Phylogenetic analysis of Dengue virus 3

**Figure 3.**
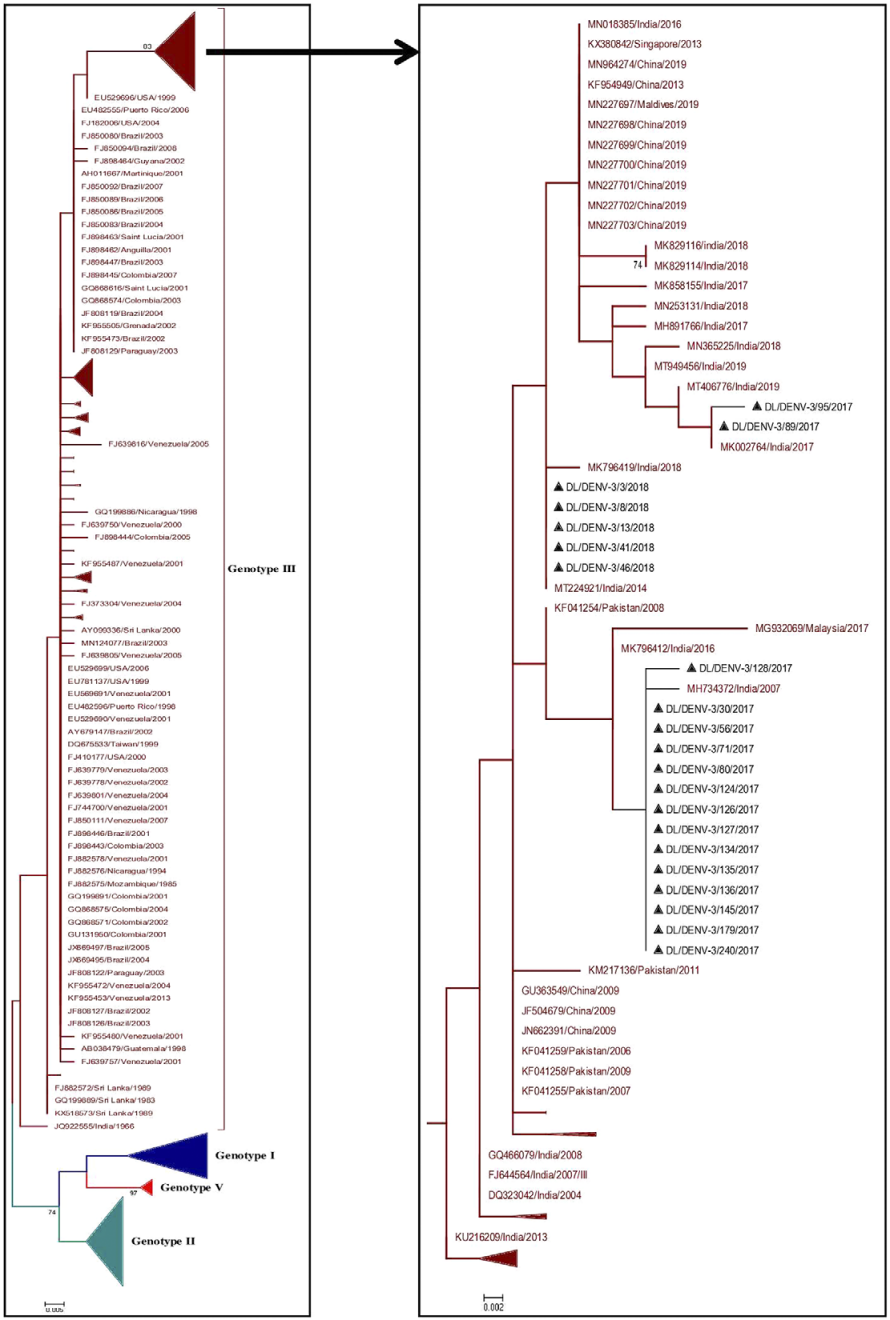
Phylogenetic tree of DENV-3 (*n* = 374). The tree was generated using the maximum likelihood approach using partial CprM gene sequences of DENV-3. The study sequences clustered in genotype III are highlighted in black with the symbol ▲. The sequences of other genotypes were collapsed as arrows for better illustration. The sequences used in the tree are shown by their accession number followed by country and the date of collection.

A total of 374 partial CprM gene sequences including 21 study sequences of Dengue virus 3 were used for the construction of a phylogenetic tree. Sixteen and 5 samples were identified as positive for DENV-3 in 2017 and 2018, respectively. The aligned region of 285 bp (95 amino acids) of the CprM region corresponds to 137 to 421 bp of the full genome of the prototype strain (H87 strain: GenBank Accession number: M93130). The identified study strains clustered with the sequences of genotype III from India, Pakistan, and China (Figure 3 and Supplementary Figure S2).

**Figure 4.**
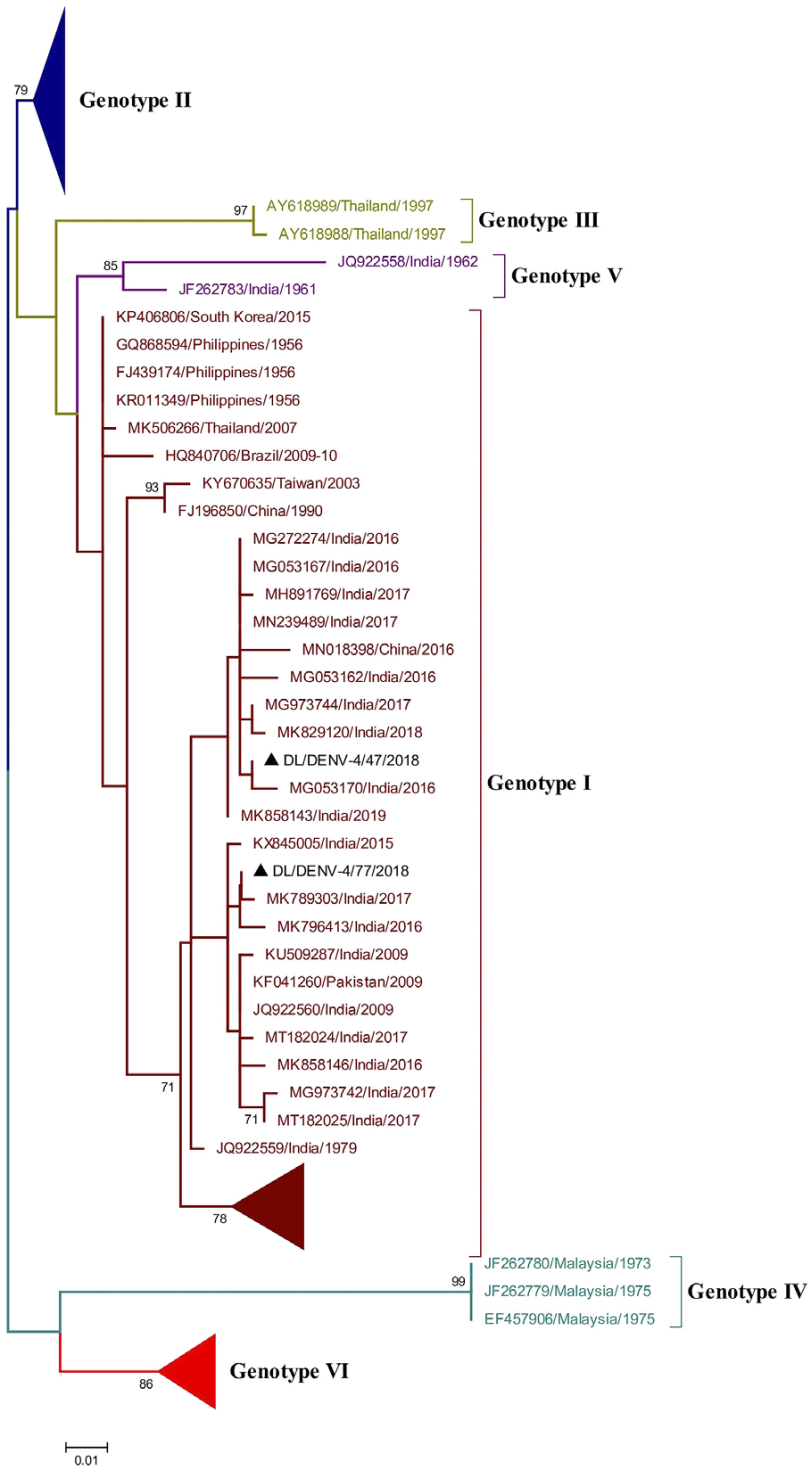
Phylogenetic tree of DENV-4 (*n* = 186). The tree was generated using the maximum likelihood approach using partial CprM gene sequences of DENV-4. The study sequences clustered in genotype I are highlighted in black with the symbol ▲. The arrows are used to provide more clarity to the illustration. The sequences used in the tree are shown by their accession number followed by country and the date of collection.

Three mutations V19A, R35K, and M108I were identified in the study strains in contrast to the full-length amino acid sequence of the prototype strain. All these mutations were also detected in earlier published sequences [14, 15]. The study strains showed nucleotide distance in the range of 6.7–7.5% and amino acid distance of 2.1% with respect to the prototype strain.

### Phylogenetic analysis of Dengue virus 4

Phylogenetic analysis of DENV-4 was conducted by aligning CprM gene sequences of the study strains with other publicly available sequences retrieved from GenBank. The alignment comprises of a total of 186 sequences including two study strains of DENV-4 identified in 2018. The aligned region corresponds to 138–479 bp (342 bp) of the full genome of the prototype strain (H241 strain: GenBank Accession number: KR011349). The study strains grouped together with the sequences of previously identified Indian strains of genotype I (Figure 4 and Supplementary Figure S3).

Three amino acid mutations were identified in the study sequences as compared to the full-length protein sequence of the prototype strain. The mutations were E18A, M102I, and V111A. These mutations were also detected in previously identified strains of DENV-4 [16, 17]. The study strains showed nucleotide distance up to 3.6% as compared to the prototype strains. The sequences showed an amino acid distance of 1.8%.

### Bayesian MCMC analysis of DENV-1

The dataset of DENV-1 strains (*n* = 170), including all its six genotypes, showed a positive temporal signal (*R* ^2^ = 0.11) (Supplementary Figure S4A) and was proceeded further for molecular clock analysis. The best-fit nucleotide substitution model for the dataset was chosen as TrN + I + G (gamma categories = 4). Strict clock and Bayesian skyline tree prior were chosen as the best-fit models in the molecular clock analysis as suggested by the Bayes factor (Table 1).

**Table 1.** Log marginal likelihoods by path sampling and stepping-stone sampling for DENV-1, DENV-3, and DENV-4

Serotype	Clock	Tree prior	Log marginal likelihood using path sampling (PS)	Log marginal likelihood using stepping stone sampling (SS)
DENV-1	Strict	Constant size	−4306.45	−4307.73
	Uncorrelated relaxed lognormal	Constant size	−4306.93	−4308.33
	Uncorrelated relaxed exponential	Constant size	−4320.00	−4321.71
	**Strict**	**Bayesian skyline**	**−4291.82**	**−4293.40**
	Uncorrelated relaxed lognormal	Bayesian skyline	−4301.43	−4303.37
	Uncorrelated relaxed exponential	Bayesian skyline	−4316.20	−4317.75
	Strict	Exponential growth	−4292.30	−4294.29
	Uncorrelated relaxed lognormal	Exponential growth	−4297.89	−4299.62
	Uncorrelated relaxed exponential	Exponential growth	−4308.65	−4310.56
DENV-3	Strict	Constant size	−2855.90	−2858.38
	Uncorrelated relaxed lognormal	Constant size	−2860.01	−2863.49
	Uncorrelated relaxed exponential	Constant size	−2857.9	−2861.26
	Strict	Bayesian skyline	−2850.03	−2852.41
	Uncorrelated relaxed lognormal	Bayesian skyline	−2852.27	−2855.46
	Uncorrelated relaxed exponential	Bayesian skyline	−2856.86	−2860.37
	**Strict**	**Exponential growth**	**−2845.30**	**−2847.42**
	Uncorrelated relaxed lognormal	Exponential growth	−2852.89	−2855.78
	Uncorrelated relaxed exponential	Exponential growth	−2852.21	−2855.47
DENV-4	Strict	Constant size	−3017.06	−3018.46
	Uncorrelated relaxed lognormal	Constant size	−3018.33	−3020.4
	Uncorrelated relaxed exponential	Constant size	−3018.65	−3019.61
	**Strict**	**Bayesian skyline**	**−3012.61**	**−3013.51**
	Uncorrelated relaxed lognormal	Bayesian skyline	−3017.87	−3019.23
	Uncorrelated relaxed exponential	Bayesian skyline	−3015.35	−3016.58
	Strict	Exponential growth	−3013.2	−3014.4
	Uncorrelated relaxed lognormal	Exponential growth	−3017.92	−3019.19
	Uncorrelated relaxed exponential	Exponential growth	−3015.74	−3017.52

**Figure 5.**
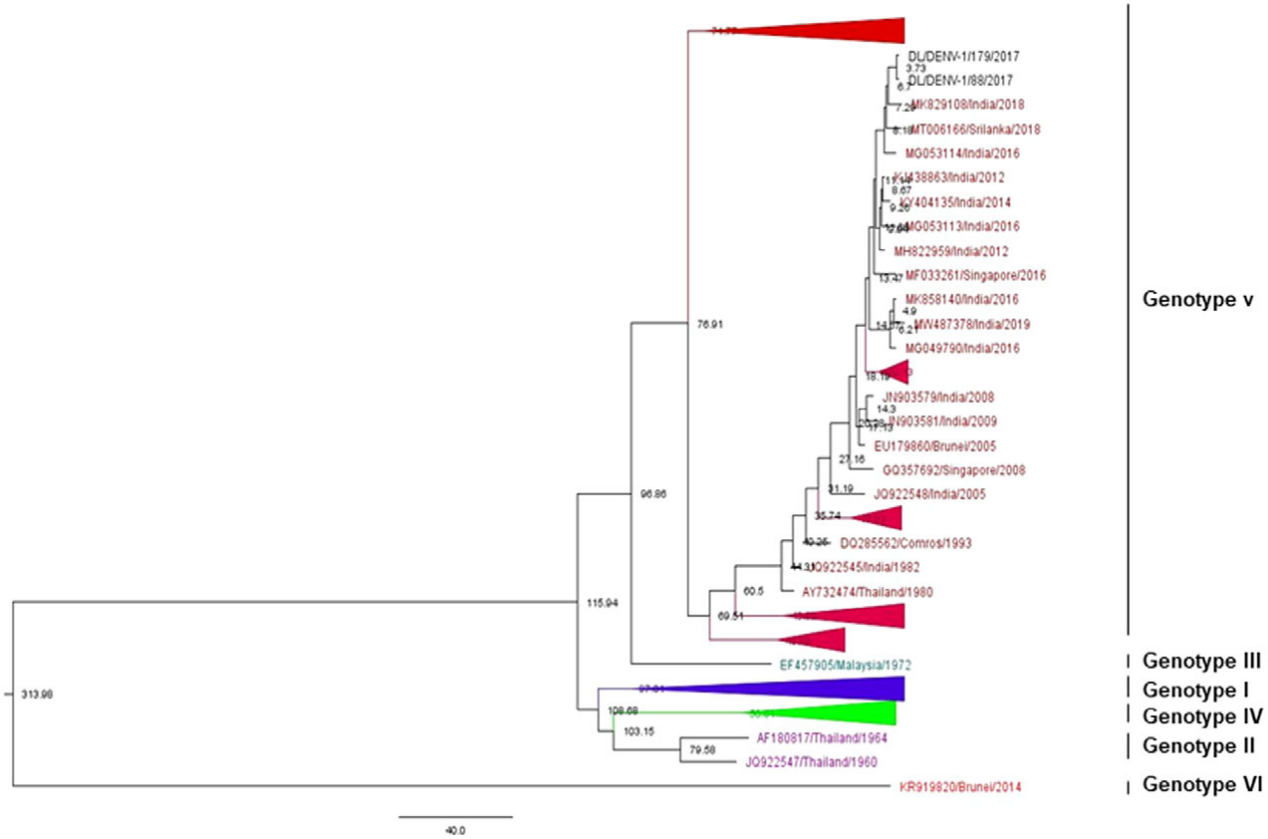
Maximum clade credibility tree of DENV-1 (*n* = 170). The tree was generated with the best-fit strict clock, Bayesian skyline model. Node ages are denoted at each node. The arrows are used to provide more clarity to the illustration.

The maximum clade credibility (MCC) tree of *n* = 170 DENV-1 sequences was generated in Fig tree v1.4.1 using the best-fit model as shown in Figure 5 (Supplementary Figure S5).

The mean nucleotide substitution rate under the strict clock was detected to be 5.99 × 10^−4^ substitutions per site per year (95% HPD (4.77 × 10^−4^–7.25 × 10^−4^ s/s/y))^.^ The age from the root was estimated to be approximately 314 years (95% HPD (229–426 years)), 1706 (95% HPD (1597–1796)). Similarly, the time to the most recent common ancestor (tMRCA) for genotype V was about 77 years (95% HPD (69–87 years)). Further, the study strains of DENV-1 clustered in genotype V were found to be approximately 4 years old (95% HPD (3–6 years)). Likewise, tMRCA for genotype I, II, and IV was estimated around 97, 80, and 59 years.

### Bayesian MCMC analysis of DENV-3

**Figure 6.**
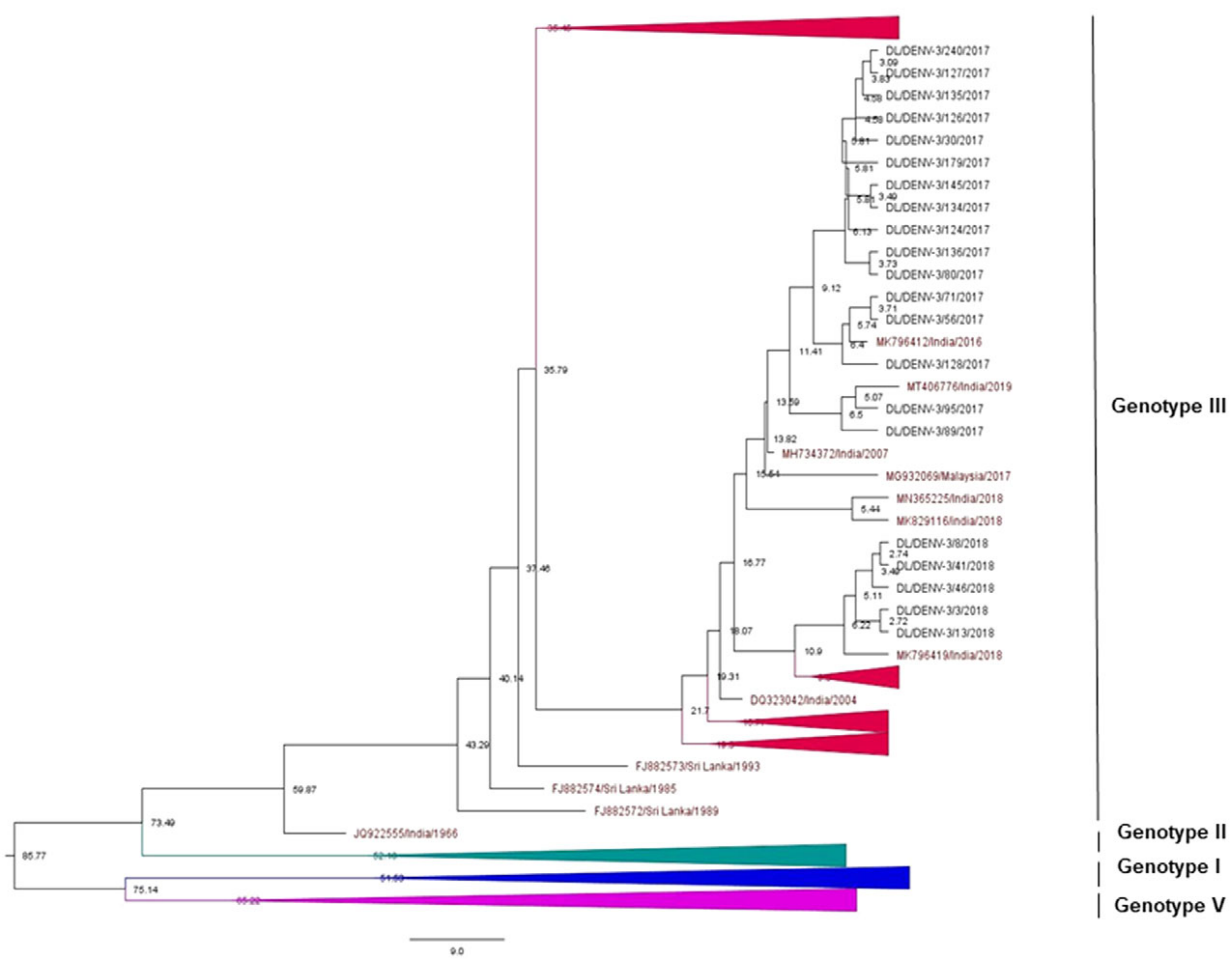
Maximum clade credibility tree of DENV-3 (*n* = 190). The tree was generated with the best-fit strict clock, exponential growth model. Node ages are denoted at each node. The arrows are used to provide more clarity to the illustration.

The dataset of DENV-3 strains (*n* = 190) was established for molecular clock analysis as it showed a sufficient temporal signal by the TempEst (*R* ^2^ = 0.31) (Supplementary Figure S4B). TrN + I + G (with gamma-distributed rate variation among sites, four categories) was selected as the best-fit nucleotide substitution model for the DENV-3 dataset. Strict clock and exponential growth tree prior were chosen as the best-fit models, suggested by the Bayes factor (Table 1). Maximum clade credibility tree of *n* = 190 DENV-3 sequences was constructed with this model as shown in Figure 6 (Supplementary Figure S6).

The mean nucleotide substitution rate under the strict clock was detected to be 7.90 × 10^−4^ substitutions per site per year (95% HPD (5.83 × 10^−4^–10.17 × 10^−4^ s/s/y)) under the best-fit model. The root of the tree diverging into four genotypes of DENV-3 (I, II, III, and V) was estimated to be 86 years old (95% HPD (72–103 years)), 1934 (95% HPD (1917–1948)). The Indian isolate JQ92255 and the isolates of DENV-3 from other countries clustered in genotype III had a mean age of about 60 years (95% HPD (54–69 years)) with almost 100% posterior probability. The mean age of the node indicating divergence of genotype III and genotype II in the best-fit model with respect to the CprM partial gene was around 74 years (95% HPD (62–88 years)). Similarly, the mean age indicating divergence between genotype I and genotype V is 75 years (95% HPD (67–86 years)). The tMRCA of genotype I and II was about 52 (95% HPD (42–62 years)) and 53 (95% HPD (47–60 years)) years, respectively. The tMRCA of genotype V was about 66 years (95% HPD (64–70 years)). The Indian lineage consisting of the study strains was shown to have an age of about 17 years (95% HPD (14–20 years)) (Figure 6).

### Bayesian MCMC analysis of DENV-4

**Figure 7.**
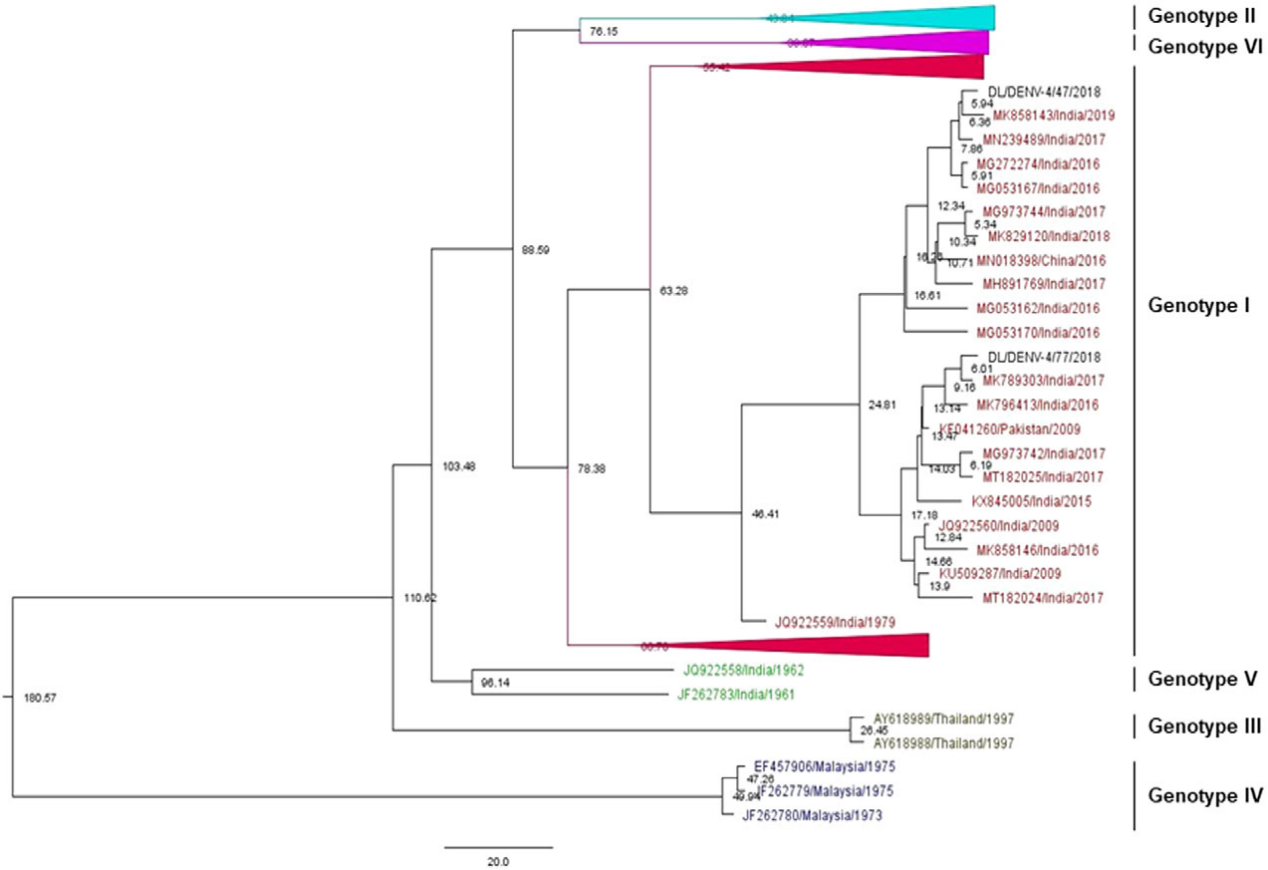
Maximum clade credibility tree of DENV-4 (*n* = 134). The tree was generated with the best-fit strict clock, Bayesian skyline model. Node ages are denoted at each node. The arrows are used to provide more clarity to the illustration.

After downsizing of the sequences in the phylogenetic tree and achieving a sufficient temporal signal in TempEst (*R* ^2^ = 0.32), the dataset of DENV-4 sequences (including 2 study strains) (*n* = 134) was selected for molecular clock analysis (Supplementary Figure S4C). The nucleotide substitution for the resulting dataset was selected as GTR + G (with gamma-distributed rate variation among sites, four categories). Strict clock and Bayesian skyline were estimated as the best-fit models for DENV-4 strains in our analysis (Table 1). The maximum clade credibility tree was constructed using the best-fit model as shown in Figure 7 (Supplementary Figure S7).

The mean evolutionary rate was estimated to be 6.23 × 10^−4^ substitutions/site/year (95% HPD (4.64 × 10^−4^–7.89 × 10^−4^ s/s/y)). The tMRCA of the tree root of all the genotypes of DENV-4 (I, II, III, IV, V, and VI) was estimated approximately as 181 years (95% HPD (133–238 years)), 1840 (95% HPD (1784–1889)). The mean age of divergence between genotype II and genotype VI is about 76 years. The analysis showed that genotype III, which included the Thailand sequences, evolved around 111 years (95% HPD (87–140 years)) ago. The date estimation showed that genotype I was introduced around 78 years ago (95% HPD (65–94 years)), that is in 1943. The age of the study sequences was found to be about 6 years old (95% HPD (3–10 years)).

The results obtained from the molecular clock analyses of the three serotypes are summarised in Table 2.

**Table 2.** Summary table of the molecular clock analysis using Bayesian MCMC approach

Serotype	Genotypes included in the study	MCC Tree sequence’s collection date range	Mean tMRCA (95% HPD) age	Mean tMRCA (95% HPD) duration	Mean nucleotide substitution rate (s/s/y) (95% HPD)
DENV-1	I, II, III, IV, V, and VI	1944–2020	314 years (229–426 years)	1706 (1597–1796)	5.99 × 10^−4^ s/s/y (4.77 × 10^−4^–7.25 × 10^−4^ s/s/y)
DENV-3	I, II, III, and V	1956–2020	86 years (72–103 years)	1934 (1917–1948)	7.90 × 10^−4^ s/s/y (5.83 × 10^−4^–10.17 × 10^−4^ s/s/y)
DENV-4	I, II, III, IV, V, and VI	1956–2021	181 years (133–238 years)	1840 (1784–1889)	6.23 × 10^−4^ s/s/y (4.64 × 10^−4^–7.89 × 10^−4^ s/s/y)

### Bayesian skyline plots

**Figure 8.**
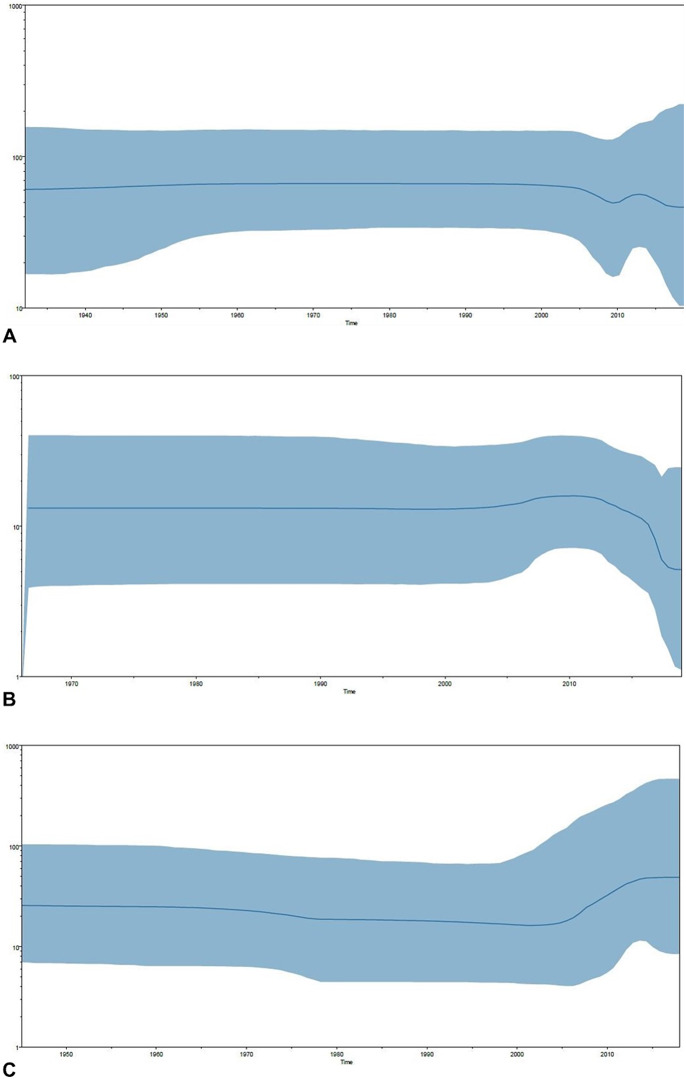
Bayesian skyline plots of Indian strains. (a) DENV-1 (*n* = 41), (b) DENV-3 (*n* = 48), and (c) DENV-4 (*n* = 23). *X*-axis – time, *Y*-axis – Neτ. The blue solid line is the median estimate of Neτ. The blue-shaded area represents 95% HPD.

On exploring the demographic history of the sampled population of DENV-1 (Indian strains *n* = 41, 1956–2019) using the Bayesian skyline plot, a constant pattern of population size was observed from 1956 to 2006 (Figure 8a). The virus population started decreasing around 2006 and reached a noticeable decline level around 2010. After 2010, the population size showed a high rise but it started declining soon around 2015. Unlike DENV-1, the population size of DENV-3 in India (Indian strains *n* = 48, 1966–2019) started increasing around 2006 and showed a significant decline around 2014–2015 (Figure 8b). The population of DENV-4 (Indian strains *n* = 23, 1961–2019) also started increasing around 2006. However, a significant rise was observed between 2010 and 2015 (Figure 8c).

### Median-joining network

**Figure 9.**
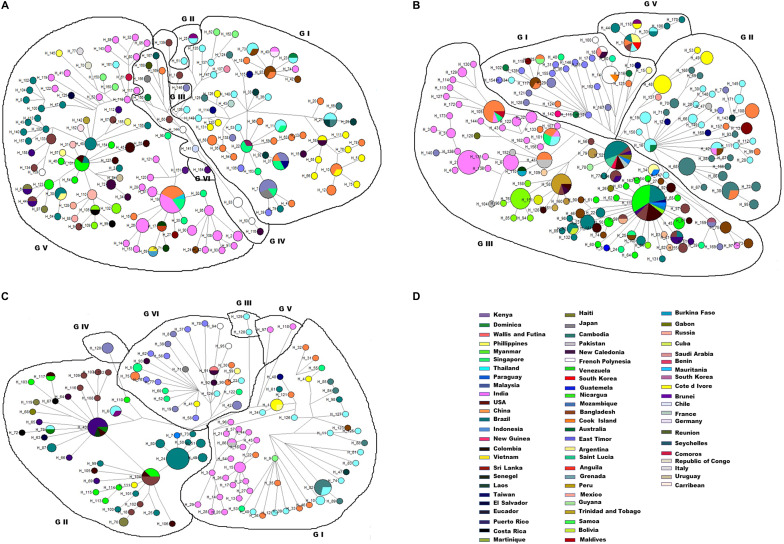
The median-joining network. (a) DENV-1, (b) DENV-3, (c) DENV-4, and (d) Countries colour-coding. The network of each serotype shows the genotypic relationships within a serotype and the pattern of clustering in each genotype. The size of the circle represents the clustering of the identical sequences collected from different geographical regions across the globe. The length of the lines does not represent any proportionality to the mutational distances amongst the strains.

The median-joining network of DENV-1 strains (*n* = 240) highlights the predominance of genotype V and genotype I of DENV-1 globally. It showed the emergence of two major haplotypes H19 and H101 of genotype V into two distinct lineages of Asian and American viral strains, respectively (Figure 9a). Most of the Indian strains including our study strains got clustered with H19, indicating closer relatedness of the Indian sequences with the Asian lineage of genotype V.

Similarly, the median-joining network of *n* = 374 sequences of DENV-3 showed evolutionary relationships in the four genotypes of DENV-3 (I, II, III, and V) (Figure 9b). The haplotype H16 from which other lineages of genotype II emerged was related to the haplotype H147 of genotype III. The nodes H75, H92, H34, H52, H56, H107, H32, and H105 of genotype III consisting of the sequences from Sri Lanka, Colombia, Argentina, Brazil, USA, India, and China are all interconnected progressively, suggesting sequential evolution of genotype III of DENV-3 from the origin. From these nodes, further minor haplotypes emerged into distinct lineages of genotype III. The study strains of DENV-3 haplotypes H2, H3, H4, H5, and H6 showed distinct branching points in the Asian lineage of genotype III.

Further, the evolution and expansion pattern of DENV-4 (*n* = 186) was also studied using median-joining network analysis. The DENV-4 network included all its six genotypes I, II, III, IV, V, and VI (Figure 9c). Genotype I represents the DENV-4 strains from Thailand, India, the Philippines, China, and Cambodia. The study isolates (H2, H3) along with the other Indian strains formed a distinct lineage in genotype I. Further, the figure also illustrates the emergence of two DENV-4 genotype II major lineages (H43 and H24) from the haplotype H104. The number of countries and their colour-coding utilised in the network analysis is given in Figure 9d.

### Selection pressure analyses

The dataset DENV-1 (*n* = 170), DENV-3 (*n* = 190), and DENV-4 (*n* = 134) were subjected to selection pressure analyses for the identification of the sites in the CprM region that are prone to mutation. The low ratio of mean dN/dS obtained by SLAC suggested negative or purifying selection in the CprM region of DENV-1, DENV-3, and DENV-4 genomes. The four codon positions of DENV-1 were detected under positive selection: 14, 16, 86, and 100. The codon position 16 was positively selected by all the four nucleotide substitution methods that were employed for the analysis. Two codon positions of DENV-3 were detected under positive selection: 19 and 79 with position 19 were positively selected by all the methods. Further, two codon positions were detected under the positive selection of DENV-4: 18 and 75. The codon position 18 was positively selected by all the above methods. The results obtained for the three DENV serotypes are summarised in Table 3.

**Table 3.** Selection pressure analyses of the CprM region of DENV-1, DENV-3, and DENV-4

Serotype	Global dN/dS	Codon positive	SLAC		FEL		MEME		FUBAR	
DENV-1	0.108		dN–dS	*p*	dN/dS	*p*	*β* ^+^	*p*	*β*–*α*	*Post. prob.*
		**14**	–	–	+∞	0.128	34.75	0.00	0.359	0.754
		15	–	–	–	–	43.48	0.00	–	–
		**16**	1.35	0.140	+∞	0.076	78.44	0.00	0.454	0.837
		19	–	–	–	–	10.17	0.21	–	–
		46	–	–	–	–	136.92	0.21	–	–
		**86**	–	–	–	–	13.71	0.19	1.379	0.735
		**100**	–	–	+∞	0.117	2.40	0.13	0.685	0.807
		105	–	–	–	–	94.67	0.25	–	–
		125	–	–	–	–	2026.88	0.00	–	–
DENV-3	0.141	**19**	3.63	0.058	+∞	0.063	6.56	0.08	2.079	0.962
		**79**	–	–	+∞	0.221	1.27	0.24	0.438	0.727
		97	–	–	–	–	–	–	2.461	0.721
		108	–	–	–	–	–	–	0.483	0.783
DENV-4	0.137	**18**	3.49	0.069	+∞	0.025	63.56	0.00	2.494	0.980
		26	–	–	–	–	10.22	0.23	–	–
		**75**	–	–	+∞	0.190	1.38	0.21	0.381	0.728
		78	–	–	–	–	80.46	0.25	–	–
		81	–	–	–	–	–	–	1.511	0.823
		102	–	–	–	–	–	–	2.367	0.877
		115	–	–	–	–	133.98	0.01	–	–

The *p*-value threshold for SLAC, FEL, and MEME was set in the range of 0.05–0.25. The posterior probability in FUBAR was set to 0.7. The sites selected by at least two methods were considered positively selected and are bold in the table. Codon positive are DENV-1: 14, 16, 86, and 100; DENV-3: 19 and 79; DENV-4: 18 and 75. The codon positions correspond to the positions of the full-length protein sequences of the respective prototype strains of DENV-1, DENV-3, and DENV-4.

### Shannon entropy analysis

**Figure 10.**
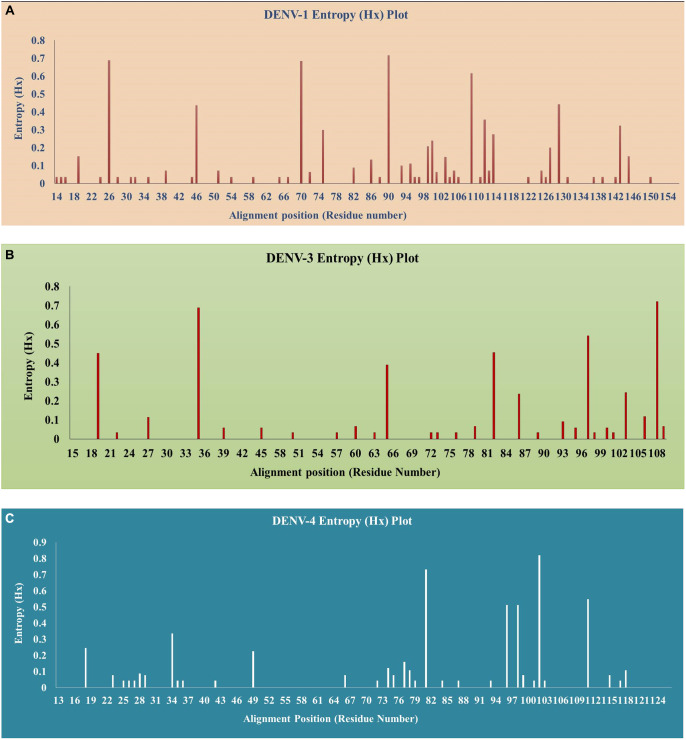
Shannon entropy plot of CprM region of Dengue virus. (a) DENV-1, (b) DENV-3, and (c) DENV-4.

The identification of the sites that are prone to mutation in the CprM region of the DENV genome was done by the entropy analysis using BioEdit (v.7.2) software for the datasets *n* = 170 (DENV-1), *n* = 190 (DENV-3), and *n* = 134 (DENV-4). For the selection of a variable site, a value of 0.2 was set as the threshold. The positions were mentioned with respect to the full-length protein sequences of the respective prototype strains of the Dengue virus. Twelve sites were identified in DENV-1 having an entropy score of more than 0.2. The sites were: 26, 46, 70, 75, 90, 99, 100, 109, 112, 114, 129, and 143. Sites 26, 70, 90, and 109 possessed an entropy score of more than 0.5 (Figure 10a). Similarly, the sites identified in DENV-3 were 19, 35, 65, 82, 86, 97, 103, and 108, amongst which 35, 97, and 108 possessed scores greater than 0.5 (Figure 10b). The analysis in the DENV-4 dataset also showed eight variable sites 18, 34, 49, 81, 96, 98, 102, and 111. The entropy score > 0.5 goes to the codon position 81, 96, 98, 102, and 111 in this conserved region of the DENV-4 genome (Figure 10c).

## Discussion

The phenomena of genetic changes in the DENV genome have profoundly affected the epidemiology and virulence of Dengue infection worldwide [18–20]. These genetic changes are crucial in tracing the origin of the disease, which is carried out principally through the evolutionary analyses of genome sequences [21]. In the past, these analyses have elucidated several important features of DENV such as rates and constraints of evolution, viral population sizes, selection pressures, and putative recombination [22]. To get better insights into the epidemic pattern of the disease, consistent monitoring in a given population is crucial, aiding in the prevention of this impending threat [23]. In this study, we have performed partial CprM gene sequence–based global phylogenetic, Bayesian MCMC, median-joining network, selection pressure, and entropy analyses. We have analysed global DENV-1, DENV-3, and DENV-4 strains, including the strains identified in our study during 2017–2018.

DENV-1 has six genotypes; Asia (I), Thailand (II), Malaysia (III), South Pacific (IV), American/African (V), and sylvatic (VI) [24, 25]. The phylogenetic analysis of DENV-1 showed clustering of the identified strains in genotype V with the previously recognised strains from India. The American-African genotype (V) of DENV-1 has been in circulation since the 1940s in India; however, the existence of the Asian genotype of DENV-1 was also reported from India in very earlier viral isolates from 1997–1998 [6]. Interestingly, another report from India had also detected the predominance of the Asian genotype (genotype I) as the cause of an epidemic in the southern part of the country in 2012 [26]. Though the Asian genotype was the cause of major outbreaks in the previous years, the clustering pattern in the phylogenetic tree of DENV-1 including our study suggested a more diverse spread of genotype V globally, particularly in the Americas and in India as well [27–30].

In the CprM phylogeny of DENV-3, four genotypes (I, II, III, and V) have been identified to date. Sequences clustered in genotype IV were not recognised by CprM gene phylogeny and hence excluded from the analyses. The phylogenetic analysis of DENV-3 revealed the clustering of the study strains into genotype III along with the sequences from China, Pakistan, and India. The clustering of study strains into different clades suggested the microevolution of DENV-3 in India during 2017–2018. This genotype of DENV-3 is under continued persistence for decades in New Delhi, India [8, 15, 31, 32]. The predominance of genotype III of DENV-3 was also reported from other geographical locations in India [33, 34]. The genotype III of DENV-3 has also spread its tentacles globally, like in Sudan, Venezuela, Saudi Arabia, and other regions of the world [35–37]. This suggested a consistent predominance of DENV-3 circulation by genotype III globally and in India as well.

Similarly, we have phylogenetically analysed the DENV-4 study strains identified in 2018. The DENV-4 study strains were grouped into genotype I along with other Indian strains. The DENV-4 genotype classification for the current study was done according to the article published in 2016 [30]. A similar report has shown the prevalence of genotype I of DENV-4 in 2016 in Pune, India. The isolates from genotype VI known as genotype IIa elsewhere may have arisen due to intra-serotypic recombination amongst independent ancestral lineages of DENV-4 [30, 38]. Some earlier and recent reports from India have also reported the occurrence of genotype I of DENV-4 [39, 40]. This genotype was also reported earlier in many other Asian countries like Cambodia, Malaysia, Philippines, Sri Lanka, Thailand, and Vietnam [41]. However, the prevalence of genotype II of DENV-4 was also identified in different parts of the world [42–44].

To determine the time-scale and evolutionary relationships of DENV, the Bayesian MCMC approach was performed using software implemented in the BEAST package [45]. The mean value of the evolutionary rates of DENV-1, DENV-3, and DENV-4 was estimated to be 5.99 × 10^−4^ substitutions/site/year (s/s/y), 7.90 × 10^−4^ s/s/y, and 6.23 × 10^−4^ s/s/y respectively. This suggested a faster rate of evolution and the existence of more recent evolutionary events in DENV-3 followed by DENV-4 and then DENV-1. Our rate estimates are similar to the previous reports published earlier on the basis of E gene phylogeny 5.58 × 10^−4^ s/s/y for DENV-1, 7.64 × 10^−4^ s/s/y for DENV-3, and, 6.72 × 10^−4^ s/s/y for DENV-4 [20, 32]. The tMRCA of DENV-1 was ≈314 years, which was similar to that reported by Pyke et al. (315 years) [25]. The prior estimate of tMRCA of DENV-1 before the assignment of genotype VI was estimated to be within the confidence limit of 89.17–170.28 years [46]. This explained the highly divergent characteristic feature of the Brun2014 strain (genotype VI) [25]. The tMRCA of the four genotypes of DENV-3 (I, II, III, and V) was estimated to be around 86 years old, which is comparable to the earlier estimates for this serotype (≈95 years) [32]. Similarly, the root age of DENV-4 was estimated to be 181 years old, indicating the existence of DENV-4 in around 1840. These estimates are comparable to the confidence limit described in earlier reports of DENV-4 (152.25–264.80 years) [46]. Further, the tMRCA of the prevalent genotypes V (77 years), III (60 years), and I (78 years) of DENV-1, DENV-3, and DENV-4 was found comparable to the estimates reported earlier, that is, 73, 63, and 84 years, respectively [20, 25, 32, 47]. This suggested more recent evolutionary changes in consistently predominating genotype III of DENV-3 in contrast to the other prevalent genotypes of the co-circulating serotypes.

Further, local evolutionary analyses were performed using Bayesian skyline plot methods to determine the population dynamics of DENV in reference to the circulation of the multiple serotypes in Indian settings. The Bayesian skyline plot of DENV-1 showed a decrease in population size in India from 2006 to 2010. A high rise after 2010 was observed, and, in the following years, the population size remained low. The decrease in the population size of DENV-1 during 2006 is in compliance with the predominance of DENV-3 during the 2006 outbreak in India [48]. This rise was followed by a stationary phase, which represents the continuous circulation of DENV-3 at a constant rate. However, a decline in the population size was observed after 2010 in DENV-3. Further, the retrospective analysis of the DENV-4 Indian population revealed a decrease in the population size of DENV-4 after 1980, which implies its low level of detection and predominance by other serotypes in India [5]. The rise in the population size of DENV-4 in the following year of 2007 is supported by the emergence of genotype I in Indian settings [5, 30, 41].

The evolutionary events and genotypic relationships were further analysed by median-joining network analyses. It was observed that genotype I and genotype V were the predominant genotypes of DENV-1 worldwide; however, the two are established in distinct regions. Genotype V is mostly predominant in American and African regions, and in India from the Asian regions. The two major nodes in genotype V, H19 and H101, have shown extensive divergence into distinct branches; however, the Asian lineage was found relatively stable compared to the one in the Americas, which further implies the phenomenon of frequent lineage replacement in the Americas [49]. Furthermore, the phenomena of replacement of DENV-1 lineages were majorly detected across heavily sampled countries like India, Puerto Rico, Brazil, Nicaragua, and Venezuela; therefore, such interpretations required more caution and consistent reports from various parts of the world.

In the network of DENV-3 strains, the Latin American isolates shared a common node, H11, with the isolates from East Africa, which was identified in the 1984–1989 outbreak in Mozambique [50]. The common ancestral node for Latin America and East Africa shares a common node with the isolates from Sri Lanka H75. This clustering pattern is supported by the earlier findings that suggested that Latin America is known to have its DENV-3 genotype III origins in East Africa due to the close relatedness amongst their identified isolates [51], Further, DENV-3 exists in the Indian subcontinent since 1966 (haplotype H97), which indicates the introduction of genotype III from the Indian subcontinent into East Africa in or before 1984 [51]. The network analysis further revealed distinct branching points for the study strains (H2, H3, H4, H5, and H6), which were identified in a span of just 2 years (2017–2018) in our investigation. This highlights the diverse nature of the nucleotide sequences of genotype III, in the Indian lineage of DENV-3.

The network of DENV-4 revealed the presence of a unique clade of Indian strains within genotype I. Isolates from India were found more closely related to the Sri Lankan isolate from 1978 (H123), suggesting it to be the progenitor of Indian viral strains [41]. The presence of a single strain from Pakistan KF041260/Pakistan in the Indian clade of genotype I of DENV-4 in our network is supported by a previous report from Sri Lanka, which suggested that the virus spread to India and/or Pakistan before re-emerging in Sri Lanka in 2009 [52]. Of the two lineages that emerged from H104, one of them (H43) caused the spread of the virus to various geographical regions like Colombia, Puerto Rico, etc., whereas the second lineage (H24) predominantly established itself in Brazil and was also the cause of the Dengue outbreak in the country during 2012–2013 [53].

In context to the selection pressure exerted over DENV-1, DENV-3, and DENV-4, a low value of mean dN/dS suggested strong purifying selection in the CprM region of the DENV genome owing to its conserved nature. Negative or purifying selection in the CprM region of the Dengue virus was also observed in earlier reports from India; however, some of the codon positions under positive selection were also detected [15, 54]. Although many sites were also identified in our study by selection pressure and entropy analyses individually, there are few detected which were selected by both methods. The codon position 100 with substitution R100K (basic polar to basic polar) was identified as positively selected with an entropy score of 0.24 in DENV-1. Similarly, a single codon position 19 with the substitution V19A (neutral non-polar to neutral non-polar) was selected by both selection pressure and entropy analyses (entropy score 0.45) in DENV-3. Further, a single codon position with substitution E18A (acidic polar to neutral non-polar) was also selected positively in DENV-4. This site also showed an entropy score (0.25) more than the threshold (0.2). All of these mutations were also identified in the sequences from earlier investigations [55–57]. These findings indicate that only in DENV-4, the mutation has caused a change in the polarity of the protein. However, the role of these mutations needs to be implicated in future studies with evolutionary analyses on a larger scale.

Phylogenetic analysis on the basis of the CprM gene region has shown to have some advantages. The region employs a single pair of primers for the amplification and sequencing of any of the four serotypes of DENV. Thus, it is more economical than E gene sequencing. Also, many previous studies have reported that the CprM gene junction can function as a tool in genotyping Dengue viruses [30, 35]. Moreover, in our study, the molecular clock estimates, that is, the tMRCA and nucleotide substitution rates, were found comparable to that of previous reports based on E gene phylogeny.

On the basis of the CprM phylogeny, we were able to decipher the various evolutionary aspects of the Dengue virus from across the globe. Though earlier studies have reported occasional outbreaks due to genotype shifts in DENV-1 (genotype V to I) [26, 58] and DENV-4 (genotype I to II) [59, 60], the prevalence and diverse nature of genotypes V and I of DENV-1 and DENV-4, respectively, remained consistent worldwide and in India as well, as highlighted in the present study by phylogenetic and network analyses. Such occurrence of outbreaks due to genotype shifts and the introduction of newly divergent strains (genotype VI in DENV-1 [25]) highlights that the emergence of the Dengue virus remains an ongoing threat. This proves that our understanding of DENV evolution is not yet extensive and its diversity to date remained uncharacterised. Nonetheless, the implication of prevalent genotypes in major Dengue epidemics and their continued persistence has the potential to cause Dengue pandemics worldwide in the future. Moreover, the faster rate of nucleotide substitution of DENV-3 compared to the other serotype warrants continued surveillance to screen the intrusions caused by the emerging serotype of DENV, so that effective and well-timed control strategies can be established to combat the future impending threat of this virus.

The main limitation of our study was that we have collected the samples from a local health centre of the university. Future studies on larger patient groups from different referral hospitals in different geographical regions are needed to elucidate the evolutionary trajectories of the Dengue virus. Such an effort of elaborate hospital and community-based surveillance from endemic regions is likely to describe the true disease burden caused by this emerging viral pathogen.

## Conclusions

The present study showed the predominant circulation of genotypes V, III, and I of DENV-1, DENV-3, and DENV-4, respectively in India and in various other parts of the world.

The CprM gene-based substitution rates and tMRCA were found comparable to the estimates of the previous studies based on E gene phylogeny. Microevolution was observed in the three serotypes in recent years; however, the study highlights the faster rate of evolution in DENV-3 compared to DENV-1 and DENV-4. Purifying selection is the driving force of evolution on the CprM gene of DENV. Genotypic and serotypic variations may lead to the emergence of new viral strains which may further lead to local evolution in new areas and may cause massive outbreaks. Hence, information concerning the genotypic composition of the circulating serotype would be beneficial in controlling the future risk imposed by the emerging viral strains of the Dengue virus.

## Supporting information

Islam et al. supplementary material Islam et al. supplementary material

Islam et al. supplementary material Islam et al. supplementary material

## Data Availability

All materials needed to replicate the findings of the article are available as Supplementary Materials.

## Supplementary material

The supplementary material for this article can be found at https://doi.org/10.1017/S0950268823000924.
