# Supplementary material for: Global and local evolutionary dynamics of Dengue virus serotypes 1, 3, and 4
Source: Epidemiol Infect. 2023 Jun 9;151:e127. doi: 10.1017/S0950268823000924 (PMC10540175; doi:10.1017/S0950268823000924)
Supplement: Islam et al. supplementary material [file S0950268823000924sup001.docx]

**Global and Local Evolutionary Dynamics of Dengue Virus Serotype 1, 3 and 4.**

**Arshi Islam ^1^, Farah Deeba ^1^, Bansidhar Tarai ^2^, Ekta Gupta ^3^, Irshad H Naqvi ^4^, Mohd. Abdullah ^4^, Ravins Dohare ^1^, Anwar Ahmed ^5^, Fahad N. Almajhdi ^5,6^, Tajamul Hussain ^5^, Shama Parveen ^1*^**

^1^ Centre for Interdisciplinary Research in Basic Sciences, Jamia Millia Islamia, New Delhi, India

^2^ Department of Microbiology and Infection Control, Max Superspeciality Hospital, New Delhi, India

^3^ Department of Clinical Virology, Institute of Liver and Biliary Sciences, New Delhi, India

^4^ Dr. M.A. Ansari Health Centre, Jamia Millia Islamia, New Delhi, India

^5^ Centre of Excellence in Biotechnology Research, College of Science, King Saud University, Riyadh, Saudi Arabia

^6^ Department of Botany & Microbiology, College of Science, King Saud University, Riyadh, Saudi Arabia

*Corresponding author. Centre for Interdisciplinary Research in Basic Sciences, Jamia Millia Islamia, New Delhi, India

E-mail: [sparveen2@jmi.ac.in](mailto:sparveen2@jmi.ac.in)

**Table S1:** Clinical and virological details of the patient’s samples collected during 2018

| **Patient Id** | **Gender** | **Age** | **Days of fever** | **Dengue virus detection**  **(serotype)** |
| --- | --- | --- | --- | --- |
| 1/18 | M | 17 | 9 | _ |
| 2/18 | M | 20 | 2 | _ |
| 3/18 | M | 20 | 2 | _+D3_ |
| 4/18 | M | 18 | 4 | _ |
| 5/18 | F | 34 | 3 | _ |
| 6/18 | M | 19 | 3 | _+D3_ |
| 7/18 | M | 20 | 3 | _ |
| 8/18 | M | 17 | 3 | _+D3_ |
| 9/18 | F | 18 | 3 | _ |
| 10/18 | F | 22 | 3 | _ |
| 11/18 | M | 22 | 2 | _ |
| 12/18 | M | 23 | 4 | _ |
| 13/18 | M | 14 | 4 | _+D3_ |
| 14/18 | M | 22 | 2 | _ |
| 15/18 | F | 28 | 2 | _ |
| 16/18 | F | 40 | 3 | _ |
| 17/18 | M | 20 | 4 | _ |
| 18/18 | M | 20 | 3 | _+D3_ |
| 19/18 | M | 17 | 2 | _+D3_ |
| 20/18 | M | 18 | 3 | _+D3_ |
| 21/18 | F | 22 | 2 | _ |
| 22/18 | F | 19 | 5 | _ |
| 23/18 | M | 18 | 7 | _ |
| 24/18 | M | 23 | 5 | _ |
| 25/18 | M | 19 | 5 | _ |
| 26/18 | F | 33 | 3 | _ |
| 27/18 | M | 21 | 3 | _+D3_ |
| 28/18 | M | 21 | 7 | _+D3_ |
| 29/18 | M | 20 | 2 | _ |
| 30/18 | M | 20 | 8 | _+D3_ |
| 31/18 | M | 15 | 2 | _ |
| 32/18 | M | 38 | 2 | _ |
| 33/18 | F | 24 | 3 | _ |
| 34/18 | M | 20 | 7 | _ |
| 35/18 | M | 21 | 7 | _+D3_ |
| 36/18 | M | 43 | 4 | _ |
| 37/18 | M | 14 | 10 | _ |
| 38/18 | F | 23 | 5 | _ |
| 39/18 | M | 22 | 4 | _ |
| 40/18 | F | 18 | 5 | _+D3_ |
| 41/18 | M | 32 | 2 | _+D3_ |
| 42/18 | M | 28 | 4 | _ |
| 43/18 | M | 24 | 3 | _+D3_ |
| 44/18 | M | 24 | 2 | _+D3_ |
| 45/18 | F | 26 | 2 | _ |
| 46/18 | M | 12 | 2 | _+D3_ |
| 47/18 | M | 35 | 3 | _+D4_ |
| 48/18 | M | 66 | 6 | _ |
| 49/18 | M | 40 | 5 | _+D3_ |
| 50/18 | F | 39 | 5 | _ |
| 51/18 | F | 25 | 2 | _+D3_ |
| 52/18 | M | 19 | 3 | _ |
| 53/18 | F | 65 | 5 | _ |
| 54/18 | F | 38 | 5 | _+D3_ |
| 55/18 | F | 50 | 6 | _+D3_ |
| 56/18 | F | 37 | 4 | _+D3_ |
| 57/18 | M | 31 | 1 | _ |
| 58/18 | M | 35 | 2 | _ |
| 59/18 | F | 38 | 7 | _+D3_ |
| 60/18 | F | 69 | 2 | _+D3_ |
| 61/18 | F | 40 | 2 | _ |
| 62/18 | F | 18 | 5 | _ |
| 63/18 | F | 12 | 4 | _ |
| 64/18 | M | 42 | 3 | _ |
| 65/18 | F | 15 | 3 | _ |
| 66/18 | M | 15 | 4 | _ |
| 67/18 | M | 20 | 3 | _ |
| 68/18 | F | 45 | 2 | _ |
| 69/18 | M | 46 | 4 | _ |
| 70/18 | F | 43 | 5 | _ |
| 71/18 | M | 37 | 2 | _ |
| 72/18 | F | 24 | 2 | _ |
| 73/18 | F | 36 | 2 | _ |
| 74/18 | M | 28 | 3 | _+D3_ |
| 75/18 | M | 51 | 5 | _+D3_ |
| 76/18 | M | 23 | 5 | _+D3_ |
| 77/18 | M | 23 | 4 | _+D4_ |
| 78/18 | F | 19 | 4 | _ |
| 79/18 | F | 20 | 3 | _ |
| 80/18 | M | 22 | 3 | _ |
| 81/18 | M | 23 | 3 | _ |
| 82/18 | M | 21 | 8 | _ |
| 83/18 | M | 22 | 3 | _ |
| 84/18 | M | 22 | 4 | _ |
| 85/18 | M | 21 | 5 | _ |
| 86/18 | M | 29 | 6 | _ |
| 87/18 | F | 55 | 4 | _ |
| 88/18 | M | 20 | 8 | _ |
| 89/18 | F | 11 | 2 | _ |

**Table S2:** Accession no. of the DENV strains identified in our study during 2017 and 2018.

| Accession no. | DENV Strain | Serotype | Genotype |
| --- | --- | --- | --- |
| MK002747 | DL/DENV-1/88/2017 | Dengue virus 1 | V |
| MK002748 | DL/DENV-1/179/2017 | Dengue virus 1 | V |
| MK002749 | DL/DENV-3/30/2017 | Dengue virus 3 | III |
| MK002750 | DL/DENV-3/56/2017 | Dengue virus 3 | III |
| MK002751 | DL/DENV-3/71/2017 | Dengue virus 3 | III |
| MK002752 | DL/DENV-3/80/2017 | Dengue virus 3 | III |
| MK002753 | DL/DENV-3/95/2017 | Dengue virus 3 | III |
| MK002754 | DL/DENV-3/124/2017 | Dengue virus 3 | III |
| MK002755 | DL/DENV-3/126/2017 | Dengue virus 3 | III |
| MK002756 | DL/DENV-3/127/2017 | Dengue virus 3 | III |
| MK002757 | DL/DENV-3/128/2017 | Dengue virus 3 | III |
| MK002758 | DL/DENV-3/134/2017 | Dengue virus 3 | III |
| MK002759 | DL/DENV-3/135/2017 | Dengue virus 3 | III |
| MK002760 | DL/DENV-3/136/2017 | Dengue virus 3 | III |
| MK002761 | DL/DENV-3/145/2017 | Dengue virus 3 | III |
| MK002762 | DL/DENV-3/179/2017 | Dengue virus 3 | III |
| MK002763 | DL/DENV-3/240/2017 | Dengue virus 3 | III |
| MK002764 | DL/DENV-3/89/2017 | Dengue virus 3 | III |
| OK173636 | DL/DENV-3/3/2018 | Dengue virus 3 | III |
| OK173637 | DL/DENV-3/8/2018 | Dengue virus 3 | III |
| OK173638 | DL/DENV-3/13/2018 | Dengue virus 3 | III |
| OK173639 | DL/DENV-3/41/2018 | Dengue virus 3 | III |
| OK173640 | DL/DENV-3/46/2018 | Dengue virus 3 | III |
| OK173641 | DL/DENV-4/47/2018 | Dengue virus 4 | I |
| OK173642 | DL/DENV-4/77/2018 | Dengue virus 4 | I |

**Table S3:** Details and selection of the sequences for evolutionary analyses of DENV-1, DENV-3 and DENV-4
**a. DENV-1**

| **Sequence detail**  **Gen Bank Accession no./Country**  **/Year of collection** | **Genotype** | **Phylogenetic analysis**  **(n=240)** | **Molecular**  **Clock**  **(n=170)** | **BSP**  **(n=41)** | **Network**  **(n=240)** | **Selection pressure**  **(n=170)** | **Entropy**  **(n=170)** |
| --- | --- | --- | --- | --- | --- | --- | --- |
| EU848545/USA/1944 | I | ✓ | ✓ | ✘ | ✓ | ✓ | ✓ |
| MK002747/India/2017 | V | ✓ | ✓ | ✓ | ✓ | ✓ | ✓ |
| MK002748/India/2017 | V | ✓ | ✓ | ✓ | ✓ | ✓ | ✓ |
| MT929529/Brazil/2015 | V | ✓ | ✓ | ✘ | ✓ | ✓ | ✓ |
| MN556094/China/2019 | I | ✓ | ✓ | ✘ | ✓ | ✓ | ✓ |
| LC428080/Vietnam/2017 | I | ✓ | ✓ | ✘ | ✓ | ✓ | ✓ |
| KT279761/Haiti/2014 | V | ✓ | ✓ | ✘ | ✓ | ✓ | ✓ |
| KX452068/Malaysia/2014 | I | ✓ | ✓ | ✘ | ✓ | ✓ | ✓ |
| KM093800/Brazil/2013 | V | ✓ | ✓ | ✘ | ✓ | ✓ | ✓ |
| JQ675358/USA/2010 | V | ✓ | ✓ | ✘ | ✓ | ✓ | ✓ |
| MW261848/China/2019 | I | ✓ | ✓ | ✘ | ✓ | ✓ | ✓ |
| MW362477/Thailand/2005 | I | ✓ | ✓ | ✘ | ✓ | ✓ | ✓ |
| MW582816/China/2019 | I | ✓ | ✓ | ✘ | ✓ | ✓ | ✓ |
| MW553080/China/2019 | I | ✓ | ✓ | ✘ | ✓ | ✓ | ✓ |
| MW487378/India/2019 | V | ✓ | ✓ | ✓ | ✓ | ✓ | ✓ |
| MW364631/New Caledonia/2014 | I | ✓ | ✓ | ✘ | ✓ | ✓ | ✓ |
| MW295817/China/2020 | V | ✓ | ✓ | ✘ | ✓ | ✓ | ✓ |
| MW288036/Senegal/2018 | V | ✓ | ✓ | ✘ | ✓ | ✓ | ✓ |
| MN886914/China/2013 | I | ✓ | ✓ | ✘ | ✓ | ✓ | ✓ |
| MW243063/Mauritania/2015 | V | ✓ | ✓ | ✘ | ✓ | ✓ | ✓ |
| MN886917/China/2006 | I | ✓ | ✓ | ✘ | ✓ | ✓ | ✓ |
| MN448696/Thailand/2012 | I | ✓ | ✓ | ✘ | ✓ | ✓ | ✓ |
| MT459980/South Korea/2011 | I | ✓ | ✓ | ✘ | ✓ | ✓ | ✓ |
| MT921569/Australia/2013 | I | ✓ | ✓ | ✘ | ✓ | ✓ | ✓ |
| MN886878/China/2014 | V | ✓ | ✘ | ✘ | ✓ | ✘ | ✘ |
| MT006166/Sri Lanka/2018 | V | ✓ | ✓ | ✘ | ✓ | ✓ | ✓ |
| MT076935/Kenya/2015 | II | ✓ | ✘ | ✘ | ✓ | ✘ | ✘ |
| MK858140/India/2016 | V | ✓ | ✓ | ✓ | ✓ | ✓ | ✓ |
| LC436668/Bangladesh/2017 | V | ✓ | ✓ | ✘ | ✓ | ✓ | ✓ |
| MN018336/Malaysia/2016 | I | ✓ | ✘ | ✘ | ✓ | ✘ | ✘ |
| MN912248/Vietnam/2017 | I | ✓ | ✘ | ✘ | ✓ | ✘ | ✘ |
| MH891771/India/2017 | V | ✓ | ✓ | ✓ | ✓ | ✓ | ✓ |
| MG877552/Laos/2012 | I | ✓ | ✓ | ✘ | ✓ | ✓ | ✓ |
| MF033261/Singapore/2016 | V | ✓ | ✓ | ✘ | ✓ | ✓ | ✓ |
| KY347013/Mexico/2014 | V | ✓ | ✓ | ✘ | ✓ | ✓ | ✓ |
| KX618706/India/2014 | V | ✓ | ✓ | ✓ | ✓ | ✓ | ✓ |
| LC335881/Japan/2014 | I | ✓ | ✘ | ✘ | ✓ | ✘ | ✘ |
| KY849720/Laos/2009 | I | ✓ | ✘ | ✘ | ✓ | ✘ | ✘ |
| KY849703/Laos/2010 | I | ✓ | ✓ | ✘ | ✓ | ✓ | ✓ |
| MF797878/Ecuador/2014 | V | ✓ | ✓ | ✘ | ✓ | ✓ | ✓ |
| KY921903/Singapore/2015 | V | ✓ | ✓ | ✘ | ✓ | ✓ | ✓ |
| KU509259/Thailand/2008 | I | ✓ | ✓ | ✘ | ✓ | ✓ | ✓ |
| KU666939/Malaysia/2014 | I | ✓ | ✓ | ✘ | ✓ | ✓ | ✓ |
| JN122281/Brazil/2011 | V | ✓ | ✓ | ✘ | ✓ | ✓ | ✓ |
| KT831765/Indonesia/2014 | I | ✓ | ✓ | ✘ | ✓ | ✓ | ✓ |
| KJ755855/India/2013 | I | ✓ | ✓ | ✓ | ✓ | ✓ | ✓ |
| KF289073/India/1956 | V | ✓ | ✓ | ✓ | ✓ | ✓ | ✓ |
| KR028435/China/2014 | I | ✓ | ✘ | ✘ | ✓ | ✘ | ✘ |
| KP686070/China/2014 | V | ✓ | ✘ | ✘ | ✓ | ✘ | ✘ |
| KJ468234/Germany/2013 | I | ✓ | ✘ | ✘ | ✓ | ✘ | ✘ |
| KJ726665/Sri Lanka/2012 | I | ✓ | ✓ | ✘ | ✓ | ✓ | ✓ |
| JQ917404/India/2009 | V | ✓ | ✘ | ✓ | ✓ | ✘ | ✘ |
| HE795086/France/2008 | I | ✓ | ✓ | ✘ | ✓ | ✓ | ✓ |
| KJ189359/Puerto Rico/2012 | V | ✓ | ✓ | ✘ | ✓ | ✓ | ✓ |
| KJ189303/Colombia/1998 | V | ✓ | ✓ | ✘ | ✓ | ✓ | ✓ |
| KF955412/Venezuela/2004 | V | ✓ | ✓ | ✘ | ✓ | ✓ | ✓ |
| KF672789/Brazil/2001 | V | ✓ | ✓ | ✘ | ✓ | ✓ | ✓ |
| KF672762/Brazil/1989 | V | ✓ | ✓ | ✘ | ✓ | ✓ | ✓ |
| JX669475/Brazil/2002 | V | ✓ | ✓ | ✘ | ✓ | ✓ | ✓ |
| JQ915075/French Polynesia/2009 | IV | ✓ | ✓ | ✘ | ✓ | ✓ | ✓ |
| JQ922547/Thailand/1960 | II | ✓ | ✓ | ✘ | ✓ | ✓ | ✓ |
| JQ922546/India/1971 | V | ✓ | ✓ | ✓ | ✓ | ✓ | ✓ |
| HM469968/Thailand/2007 | I | ✓ | ✓ | ✘ | ✓ | ✓ | ✓ |
| JQ045563/USA/2010 | V | ✓ | ✘ | ✘ | ✓ | ✘ | ✘ |
| GU056030/Venezuela/1997 | V | ✓ | ✓ | ✘ | ✓ | ✓ | ✓ |
| GQ868568/Colombia/2007 | V | ✓ | ✓ | ✘ | ✓ | ✓ | ✓ |
| GQ868635/Cambodia/2008 | I | ✓ | ✓ | ✘ | ✓ | ✓ | ✓ |
| FJ410190/USA/1987 | V | ✓ | ✓ | ✘ | ✓ | ✓ | ✓ |
| EU677157/Vietnam/2007 | I | ✓ | ✓ | ✘ | ✓ | ✓ | ✓ |
| JQ048541/China/2011 | I | ✓ | ✓ | ✘ | ✓ | ✓ | ✓ |
| GQ398255/Singapore/2008 | I | ✓ | ✘ | ✘ | ✓ | ✘ | ✘ |
| DQ672563/USA/2001 | IV | ✓ | ✓ | ✘ | ✓ | ✓ | ✓ |
| DQ285562 /Comoros/1993 | V | ✓ | ✓ | ✘ | ✓ | ✓ | ✓ |
| AY726555/Myanmar/1998 | I | ✓ | ✓ | ✘ | ✓ | ✓ | ✓ |
| MN869909/China/2018 | I | ✓ | ✓ | ✘ | ✓ | ✓ | ✓ |
| MT929571/Brazil/2019 | V | ✓ | ✓ | ✘ | ✓ | ✓ | ✓ |
| LC428070/Vietnam/2017 | I | ✓ | ✓ | ✘ | ✓ | ✓ | ✓ |
| LC428068/Vietnam/2017 | I | ✓ | ✓ | ✘ | ✓ | ✓ | ✓ |
| KX452060/Malaysia/2014 | I | ✓ | ✘ | ✘ | ✓ | ✘ | ✘ |
| KC692517/Argentina/2010 | V | ✓ | ✓ | ✘ | ✓ | ✓ | ✓ |
| KC692510/Argentina/2009 | V | ✓ | ✘ | ✘ | ✓ | ✘ | ✘ |
| KF971870/China/2013 | I | ✓ | ✓ | ✘ | ✓ | ✓ | ✓ |
| KF971869/China/2013 | I | ✓ | ✘ | ✘ | ✓ | ✘ | ✘ |
| MW261831/China/2019 | I | ✓ | ✘ | ✘ | ✓ | ✘ | ✘ |
| MW243052/CotedIvoire/2019 | V | ✓ | ✓ | ✘ | ✓ | ✓ | ✓ |
| MW243050/Burkina Faso/2017 | V | ✓ | ✘ | ✘ | ✓ | ✘ | ✘ |
| MW243006/Republic of the Congo/2016 | V | ✓ | ✓ | ✘ | ✓ | ✓ | ✓ |
| MW243062/Senegal/2019 | V | ✓ | ✓ | ✘ | ✓ | ✓ | ✓ |
| MW208056/Brazil/2019 | V | ✓ | ✘ | ✘ | ✓ | ✘ | ✘ |
| MN449013/Ecuador/2014 | V | ✓ | ✓ | ✘ | ✓ | ✓ | ✓ |
| MN448631/Thailand/2009 | I | ✓ | ✓ | ✘ | ✓ | ✓ | ✓ |
| MN448597/Thailand/2009 | I | ✓ | ✘ | ✘ | ✓ | ✘ | ✘ |
| MK588396/India/2016 | V | ✓ | ✘ | ✓ | ✓ | ✘ | ✘ |
| MT126441/India/2017 | I | ✓ | ✓ | ✓ | ✓ | ✓ | ✓ |
| MN960677/China/2019 | I | ✓ | ✘ | ✘ | ✓ | ✘ | ✘ |
| MN018333/Vietnam/2016 | I | ✓ | ✓ | ✘ | ✓ | ✓ | ✓ |
| MN018314/Malaysia/2016 | I | ✓ | ✘ | ✘ | ✓ | ✘ | ✘ |
| MN018308/China/2014 | V | ✓ | ✘ | ✘ | ✓ | ✘ | ✘ |
| MN912158/Vietnam/2017 | I | ✓ | ✓ | ✘ | ✓ | ✓ | ✓ |
| MN577472/Italy/2019 | V | ✓ | ✓ | ✘ | ✓ | ✓ | ✓ |
| MG840578/China/2017 | I | ✓ | ✓ | ✘ | ✓ | ✓ | ✓ |
| MG840573/Malaysia/2016 | I | ✓ | ✘ | ✘ | ✓ | ✘ | ✘ |
| MG840561/Brazil/2016 | I | ✓ | ✓ | ✘ | ✓ | ✓ | ✓ |
| MN600714/Benin/2019 | V | ✓ | ✘ | ✘ | ✓ | ✘ | ✘ |
| MH891767/India/2017 | V | ✓ | ✘ | ✓ | ✓ | ✘ | ✘ |
| MK829130/India/2018 | V | ✓ | ✓ | ✓ | ✓ | ✓ | ✓ |
| MK829129/India/2018 | V | ✓ | ✓ | ✓ | ✓ | ✓ | ✓ |
| MK829108/India/2018 | V | ✓ | ✓ | ✓ | ✓ | ✓ | ✓ |
| MG181997/French Polynesia/2008 | IV | ✓ | ✓ | ✘ | ✓ | ✓ | ✓ |
| MK994544/India/2018 | V | ✓ | ✘ | ✓ | ✓ | ✘ | ✘ |
| MK796420/India/2018 | V | ✓ | ✘ | ✓ | ✓ | ✘ | ✘ |
| MK796417/India/2018 | V | ✓ | ✘ | ✓ | ✓ | ✘ | ✘ |
| MH708485/Thailand/2012 | I | ✓ | ✘ | ✘ | ✓ | ✘ | ✘ |
| MH349375/Uruguay/2016 | V | ✓ | ✓ | ✘ | ✓ | ✓ | ✓ |
| MG721063/India/2016 | V | ✓ | ✓ | ✓ | ✓ | ✓ | ✓ |
| MG721060/India/2016 | V | ✓ | ✘ | ✓ | ✓ | ✘ | ✘ |
| MH822959/India/2012 | V | ✓ | ✓ | ✓ | ✓ | ✓ | ✓ |
| MH822958/India/2012 | V | ✓ | ✘ | ✓ | ✓ | ✘ | ✘ |
| MH557860/India/2016 | V | ✓ | ✘ | ✓ | ✓ | ✘ | ✘ |
| MG877555/New Caledonia/2013 | I | ✓ | ✘ | ✘ | ✓ | ✘ | ✘ |
| MG877553/Haiti/2012 | V | ✓ | ✓ | ✘ | ✓ | ✓ | ✓ |
| MF033258 /Singapore/2016 | I | ✓ | ✘ | ✘ | ✓ | ✘ | ✘ |
| KY347002/Mexico/2012 | V | ✓ | ✓ | ✘ | ✓ | ✓ | ✓ |
| KX618705/India/2014 | V | ✓ | ✓ | ✓ | ✓ | ✓ | ✓ |
| MF314188/Singapore/2016 | I | ✓ | ✘ | ✘ | ✓ | ✘ | ✘ |
| MG053117/India/2016 | V | ✓ | ✘ | ✓ | ✓ | ✘ | ✘ |
| MG053114/India/2016 | V | ✓ | ✓ | ✓ | ✓ | ✓ | ✓ |
| MG053113/India/2016 | V | ✓ | ✓ | ✓ | ✓ | ✓ | ✓ |
| KY926849/French Polynesia/2008 | IV | ✓ | ✘ | ✘ | ✓ | ✘ | ✘ |
| KY849704/Laos/2011 | I | ✓ | ✓ | ✘ | ✓ | ✓ | ✓ |
| KY474307/Ecuador/2014 | V | ✓ | ✘ | ✘ | ✓ | ✘ | ✘ |
| KY404135/India/2014 | V | ✓ | ✓ | ✓ | ✓ | ✓ | ✓ |
| KT827379/China/2015 | I | ✓ | ✓ | ✘ | ✓ | ✓ | ✓ |
| KT827377/China/2014 | V | ✓ | ✘ | ✘ | ✓ | ✘ | ✘ |
| KT827370/China/2013 | I | ✓ | ✓ | ✘ | ✓ | ✓ | ✓ |
| KT827368/China/2007 | I | ✓ | ✓ | ✘ | ✓ | ✓ | ✓ |
| KT827365/China/2006 | I | ✓ | ✓ | ✘ | ✓ | ✓ | ✓ |
| GQ357692/Singapore/2008 | V | ✓ | ✓ | ✘ | ✓ | ✓ | ✓ |
| JF960211/Singapore/2009 | V | ✓ | ✘ | ✘ | ✓ | ✘ | ✘ |
| KJ806953/Singapore/2013 | I | ✓ | ✘ | ✘ | ✓ | ✘ | ✘ |
| LC016760/Japan/2014 | I | ✓ | ✘ | ✘ | ✓ | ✘ | ✘ |
| KX544823/Costa Rica/2015 | V | ✓ | ✓ | ✘ | ✓ | ✓ | ✓ |
| JN122280/Brazil/1986 | V | ✓ | ✘ | ✘ | ✓ | ✘ | ✘ |
| HM450105/Brazil/2002 | V | ✓ | ✓ | ✘ | ✓ | ✓ | ✓ |
| HM450103/Brazil/2007 | V | ✓ | ✓ | ✘ | ✓ | ✓ | ✓ |
| HM450101/Brazil/2007 | V | ✓ | ✓ | ✘ | ✓ | ✓ | ✓ |
| HM450087/Brazil/2000 | V | ✓ | ✓ | ✘ | ✓ | ✓ | ✓ |
| HQ026760/Brazil/1986 | V | ✓ | ✘ | ✘ | ✓ | ✘ | ✘ |
| HM043710/Brazil/2009 | V | ✓ | ✓ | ✘ | ✓ | ✓ | ✓ |
| KX380806/Singapore/2013 | V | ✓ | ✘ | ✘ | ✓ | ✘ | ✘ |
| KU365900/Taiwan/2014 | I | ✓ | ✘ | ✘ | ✓ | ✘ | ✘ |
| KR024707/China/2014 | V | ✓ | ✘ | ✘ | ✓ | ✘ | ✘ |
| KJ534633/Costa Rica/2012 | V | ✓ | ✓ | ✘ | ✓ | ✓ | ✓ |
| KC762654/Indonesia/2007 | I | ✓ | ✓ | ✘ | ✓ | ✓ | ✓ |
| KP398852/Sri Lanka/2014 | I | ✓ | ✘ | ✘ | ✓ | ✘ | ✘ |
| KJ649286/Saudi Arabia/2011 | I | ✓ | ✓ | ✘ | ✓ | ✓ | ✓ |
| KJ438863/India/2012 | V | ✓ | ✓ | ✓ | ✓ | ✓ | ✓ |
| KF973462/Nicaragua/2011 | V | ✓ | ✓ | ✘ | ✓ | ✓ | ✓ |
| KF289072/India/2011 | V | ✓ | ✘ | ✓ | ✓ | ✘ | ✘ |
| KJ420624/India/2011 | V | ✓ | ✘ | ✓ | ✓ | ✘ | ✘ |
| JQ692085/India/2010 | V | ✓ | ✘ | ✓ | ✓ | ✘ | ✘ |
| KJ189338/Mexico/2008 | V | ✓ | ✓ | ✘ | ✓ | ✓ | ✓ |
| KJ189304/Colombia/2005 | V | ✓ | ✘ | ✘ | ✓ | ✘ | ✘ |
| KF955446/Viet Nam/2008 | I | ✓ | ✓ | ✘ | ✓ | ✓ | ✓ |
| KF955439/Puerto Rico/1995 | V | ✓ | ✓ | ✘ | ✓ | ✓ | ✓ |
| KF672776/Brazil/1998 | V | ✓ | ✓ | ✘ | ✓ | ✓ | ✓ |
| AB608789/Taiwan/1994 | I | ✓ | ✓ | ✘ | ✓ | ✓ | ✓ |
| AB608787/Taiwan/2008 | I | ✓ | ✓ | ✘ | ✓ | ✓ | ✓ |
| JQ915080/New Caledonia/2010 | IV | ✓ | ✓ | ✘ | ✓ | ✓ | ✓ |
| JQ922548/India/2005 | V | ✓ | ✓ | ✓ | ✓ | ✓ | ✓ |
| JQ922545/India/1982 | V | ✓ | ✓ | ✓ | ✓ | ✓ | ✓ |
| JQ922544/India/1963 | V | ✓ | ✓ | ✓ | ✓ | ✓ | ✓ |
| JN903581/India/2009 | V | ✓ | ✓ | ✓ | ✓ | ✓ | ✓ |
| JN903579/India/2008 | V | ✓ | ✓ | ✓ | ✓ | ✓ | ✓ |
| JN903578/India/2007 | V | ✓ | ✓ | ✓ | ✓ | ✓ | ✓ |
| JN819425/Venezuela/2004 | V | ✓ | ✓ | ✘ | ✓ | ✓ | ✓ |
| JN819423/Cambodia/2001 | I | ✓ | ✓ | ✘ | ✓ | ✓ | ✓ |
| JN819417/El Salvador/1993 | V | ✓ | ✓ | ✘ | ✓ | ✓ | ✓ |
| GU131772/Vietnam/2008 | I | ✓ | ✓ | ✘ | ✓ | ✓ | ✓ |
| GQ868561/Colombia/1999 | V | ✓ | ✘ | ✘ | ✓ | ✘ | ✘ |
| GQ868560/Colombia/1998 | V | ✓ | ✓ | ✘ | ✓ | ✓ | ✓ |
| GQ868521/Mexico/2007 | V | ✓ | ✓ | ✘ | ✓ | ✓ | ✓ |
| GQ868639/Cambodia/2006 | I | ✓ | ✓ | ✘ | ✓ | ✓ | ✓ |
| FJ898390/Vietnam/2005 | I | ✓ | ✓ | ✘ | ✓ | ✓ | ✓ |
| FJ898371/Vietnam/2007 | I | ✓ | ✓ | ✘ | ✓ | ✓ | ✓ |
| FJ810419/Nicaragua/2006 | V | ✓ | ✓ | ✘ | ✓ | ✓ | ✓ |
| FJ810415/Venezuela/2005 | V | ✓ | ✘ | ✘ | ✓ | ✘ | ✘ |
| FJ639794/Venezuela/2004 | V | ✓ | ✘ | ✘ | ✓ | ✘ | ✘ |
| FJ562106/USA/1986 | V | ✓ | ✓ | ✘ | ✓ | ✓ | ✓ |
| FJ562105/USA/1993 | V | ✓ | ✓ | ✘ | ✓ | ✓ | ✓ |
| FJ562104/Nicaragua/2006 | V | ✓ | ✘ | ✘ | ✓ | ✘ | ✘ |
| FJ547087/USA/1992 | V | ✓ | ✘ | ✘ | ✓ | ✘ | ✘ |
| JN638344/Thailand/1995 | I | ✓ | ✓ | ✘ | ✓ | ✓ | ✓ |
| JN638343/Thailand/1992 | I | ✓ | ✓ | ✘ | ✓ | ✓ | ✓ |
| JN638342/Thailand/1990 | I | ✓ | ✓ | ✘ | ✓ | ✓ | ✓ |
| JN697058/Malaysia/2005 | I | ✓ | ✓ | ✘ | ✓ | ✓ | ✓ |
| HQ891316/Sri Lanka/2009 | I | ✓ | ✘ | ✘ | ✓ | ✘ | ✘ |
| AB609588/USA/1944 | I | ✓ | ✓ | ✘ | ✓ | ✓ | ✓ |
| AB519681/Brazil/2001 | V | ✓ | ✓ | ✘ | ✓ | ✓ | ✓ |
| GU370048/Singapore/2008 | I | ✓ | ✘ | ✘ | ✓ | ✘ | ✘ |
| EU863650/Chile/2002 | IV | ✓ | ✓ | ✘ | ✓ | ✓ | ✓ |
| AY079173/Peru/2002 | V | ✓ | ✓ | ✘ | ✓ | ✓ | ✓ |
| EF127001/India/2006 | V | ✓ | ✓ | ✓ | ✓ | ✓ | ✓ |
| DQ672559/French Polynesia/2001 | IV | ✓ | ✓ | ✘ | ✓ | ✓ | ✓ |
| AF298807/CotedIvoire/1998 | V | ✓ | ✓ | ✘ | ✓ | ✓ | ✓ |
| EF457905/Malaysia/1972 | III | ✓ | ✓ | ✘ | ✓ | ✓ | ✓ |
| AY732483/Thailand/1981 | I | ✓ | ✓ | ✘ | ✓ | ✓ | ✓ |
| AY732482/Thailand/2001 | I | ✓ | ✓ | ✘ | ✓ | ✓ | ✓ |
| AY732480/Thailand/1994 | I | ✓ | ✓ | ✘ | ✓ | ✓ | ✓ |
| AY722801/Myanmar/1976 | V | ✓ | ✓ | ✘ | ✓ | ✓ | ✓ |
| EU179860/Brunei/2005 | V | ✓ | ✓ | ✘ | ✓ | ✓ | ✓ |
| D00501/Caribbean/1977 | V | ✓ | ✓ | ✘ | ✓ | ✓ | ✓ |
| MG049790/India/2016 | V | ✓ | ✓ | ✓ | ✓ | ✓ | ✓ |
| GU131837/Venezuela/2005 | V | ✓ | ✘ | ✘ | ✓ | ✘ | ✘ |
| FJ873809/Venezuela/2007 | V | ✓ | ✘ | ✘ | ✓ | ✘ | ✘ |
| FJ639823/Venezuela/2006 | V | ✓ | ✓ | ✘ | ✓ | ✓ | ✓ |
| EU596504/Nicaragua/2005 | V | ✓ | ✘ | ✘ | ✓ | ✘ | ✘ |
| HQ166037/Mexico/2008 | V | ✓ | ✘ | ✘ | ✓ | ✘ | ✘ |
| KJ189367/Puerto Rico/2010 | V | ✓ | ✘ | ✘ | ✓ | ✘ | ✘ |
| AY277659/Argentina/2000 | V | ✓ | ✓ | ✘ | ✓ | ✓ | ✓ |
| AF226685/Brazil/1990 | V | ✓ | ✓ | ✘ | ✓ | ✓ | ✓ |
| AF311958/Brazil/1997 | V | ✓ | ✓ | ✘ | ✓ | ✓ | ✓ |
| FJ547086/Puerto Rico/1995 | V | ✓ | ✓ | ✘ | ✓ | ✓ | ✓ |
| AF226687/French Guiana/1989 | V | ✓ | ✓ | ✘ | ✓ | ✓ | ✓ |
| AY722803/Myanmar/1998 | V | ✓ | ✓ | ✘ | ✓ | ✓ | ✓ |
| AY732474/Thailand/1980 | V | ✓ | ✓ | ✘ | ✓ | ✓ | ✓ |
| AF180817/Thailand/1964 | II | ✓ | ✓ | ✘ | ✓ | ✓ | ✓ |
| AY713475/Myanmar/2001 | I | ✓ | ✓ | ✘ | ✓ | ✓ | ✓ |
| FJ469909/Singapore/2003 | I | ✓ | ✘ | ✘ | ✓ | ✘ | ✘ |
| HM469966/Thailand/2007 | I | ✓ | ✘ | ✘ | ✓ | ✘ | ✘ |
| EU081281/Singapore/2006 | I | ✓ | ✓ | ✘ | ✓ | ✓ | ✓ |
| KF887994/Thailand/2013 | I | ✓ | ✘ | ✘ | ✓ | ✘ | ✘ |
| HM488255/Cambodia/2007 | I | ✓ | ✘ | ✘ | ✓ | ✘ | ✘ |
| HQ891315/Sri Lanka/2009 | I | ✓ | ✘ | ✘ | ✓ | ✘ | ✘ |
| KR919820/Brunei/2014 | VI | ✓ | ✓ | ✘ | ✓ | ✓ | ✓ |
| MH921567/Australia/2016 | IV | ✓ | ✓ | ✘ | ✓ | ✓ | ✓ |
| AB074761/Japan/1988 | IV | ✓ | ✓ | ✘ | ✓ | ✓ | ✓ |
| DQ285561/Seychelles/2004 | IV | ✓ | ✓ | ✘ | ✓ | ✓ | ✓ |
| DQ285560/Reunion/2004 | IV | ✓ | ✓ | ✘ | ✓ | ✓ | ✓ |
| AY376738/China/1999 | IV | ✓ | ✓ | ✘ | ✓ | ✓ | ✓ |

**b. DENV-3**

| **Sequence detail**  **Gen Bank Accession no./Country**  **/Year of collection** | **Genotype** | **Phylogenetic analysis**  **(n=374)** | **Molecular clock**  **(n=190)** | **BSP**  **(n=48)** | **Network**  **(n=374)** | **Selection pressure**  **(n=190)** | **Entropy**  **(n=190)** |
| --- | --- | --- | --- | --- | --- | --- | --- |
| M93130/Philippines/1956 | V | ✓ | ✓ | ✘ | ✓ | ✓ | ✓ |
| MK002749/India/2017 | III | ✓ | ✓ | ✓ | ✓ | ✓ | ✓ |
| MK002750/India/2017 | III | ✓ | ✓ | ✓ | ✓ | ✓ | ✓ |
| MK002751/India/2017 | III | ✓ | ✓ | ✓ | ✓ | ✓ | ✓ |
| MK002752/India/2017 | III | ✓ | ✓ | ✓ | ✓ | ✓ | ✓ |
| MK002753/India/2017 | III | ✓ | ✓ | ✓ | ✓ | ✓ | ✓ |
| MK002754/India/2017 | III | ✓ | ✓ | ✓ | ✓ | ✓ | ✓ |
| MK002755/India/2017 | III | ✓ | ✓ | ✓ | ✓ | ✓ | ✓ |
| MK002756/India/2017 | III | ✓ | ✓ | ✓ | ✓ | ✓ | ✓ |
| MK002757/India/2017 | III | ✓ | ✓ | ✓ | ✓ | ✓ | ✓ |
| MK002758/India/2017 | III | ✓ | ✓ | ✓ | ✓ | ✓ | ✓ |
| MK002759/India/2017 | III | ✓ | ✓ | ✓ | ✓ | ✓ | ✓ |
| MK002760/India/2017 | III | ✓ | ✓ | ✓ | ✓ | ✓ | ✓ |
| MK002761/India/2017 | III | ✓ | ✓ | ✓ | ✓ | ✓ | ✓ |
| MK002762/India/2017 | III | ✓ | ✓ | ✓ | ✓ | ✓ | ✓ |
| MK002763/India/2017 | III | ✓ | ✓ | ✓ | ✓ | ✓ | ✓ |
| MK002764/India/2017 | III | ✓ | ✓ | ✓ | ✓ | ✓ | ✓ |
| OK173636/India/2018 | III | ✓ | ✓ | ✓ | ✓ | ✓ | ✓ |
| OK173637/India/2018 | III | ✓ | ✓ | ✓ | ✓ | ✓ | ✓ |
| OK173638/India/2018 | III | ✓ | ✓ | ✓ | ✓ | ✓ | ✓ |
| OK173639/India/2018 | III | ✓ | ✓ | ✓ | ✓ | ✓ | ✓ |
| OK173640/India/2018 | III | ✓ | ✓ | ✓ | ✓ | ✓ | ✓ |
| AB010986/Malaysia/1993 | II | ✓ | ✓ | ✘ | ✓ | ✓ | ✓ |
| AB038479/Guatemala/1998 | III | ✓ | ✓ | ✘ | ✓ | ✓ | ✓ |
| DQ675520/Indonesia/1998 | II | ✓ | ✓ | ✘ | ✓ | ✓ | ✓ |
| DQ675519/Taiwan/1995 | I | ✓ | ✓ | ✘ | ✓ | ✓ | ✓ |
| DQ675533/Taiwan/1999 | III | ✓ | ✓ | ✘ | ✓ | ✓ | ✓ |
| DQ675531/Taiwan/1998 | II | ✓ | ✓ | ✘ | ✓ | ✓ | ✓ |
| AY496871/Bangladesh/2002 | II | ✓ | ✓ | ✘ | ✓ | ✓ | ✓ |
| AY744684/French Polynesia/1992 | I | ✓ | ✓ | ✘ | ✓ | ✓ | ✓ |
| AY744681/French Polynesia/1990 | I | ✓ | ✘ | ✘ | ✓ | ✘ | ✘ |
| AY744680/French Polynesia/1990 | I | ✓ | ✘ | ✘ | ✓ | ✘ | ✘ |
| AY744678/French Polynesia/1989 | I | ✓ | ✘ | ✘ | ✓ | ✘ | ✘ |
| AY496879/Philippines/1997 | I | ✓ | ✓ | ✘ | ✓ | ✓ | ✓ |
| AY496873/Bangladesh/2002 | II | ✓ | ✘ | ✘ | ✓ | ✘ | ✘ |
| AY923865/Thailand/1994 | II | ✓ | ✓ | ✘ | ✓ | ✓ | ✓ |
| DQ401690/Indonesia/1982 | I | ✓ | ✓ | ✘ | ✓ | ✓ | ✓ |
| AY496877/Bangladesh/2002 | II | ✓ | ✘ | ✘ | ✓ | ✘ | ✘ |
| AY744685/French Polynesia/1994 | I | ✓ | ✓ | ✘ | ✓ | ✓ | ✓ |
| AY676353/Thailand/1987 | II | ✓ | ✓ | ✘ | ✓ | ✓ | ✓ |
| AY676351/Thailand/1993 | II | ✓ | ✓ | ✘ | ✓ | ✓ | ✓ |
| AY676349/Thailand/1998 | II | ✓ | ✘ | ✘ | ✓ | ✘ | ✘ |
| EF629370/Brazil/2002 | V | ✓ | ✘ | ✘ | ✓ | ✘ | ✘ |
| EF629369/Brazil/2002 | III | ✓ | ✓ | ✘ | ✓ | ✓ | ✓ |
| EF629368/Brazil/2004 | III | ✓ | ✓ | ✘ | ✓ | ✓ | ✓ |
| EF643017/Brazil/2003 | III | ✓ | ✓ | ✘ | ✓ | ✓ | ✓ |
| EU081197/Singapore/2005 | III | ✓ | ✓ | ✘ | ✓ | ✓ | ✓ |
| EU081181/Singapore/2004 | III | ✓ | ✓ | ✘ | ✓ | ✓ | ✓ |
| AY744679/French Polynesia/1990 | I | ✓ | ✘ | ✘ | ✓ | ✘ | ✘ |
| AY679147/Brazil/2002 | III | ✓ | ✘ | ✘ | ✓ | ✘ | ✘ |
| AY099337/Martinique/1999 | III | ✓ | ✓ | ✘ | ✓ | ✓ | ✓ |
| AY099336/Sri Lanka/2000 | III | ✓ | ✓ | ✘ | ✓ | ✓ | ✓ |
| AF317645/China/1980 | V | ✓ | ✘ | ✘ | ✓ | ✘ | ✘ |
| AY744683/French Polynesia/1992 | I | ✓ | ✘ | ✘ | ✓ | ✘ | ✘ |
| HG316484/Thailand/2010 | II | ✓ | ✓ | ✘ | ✓ | ✓ | ✓ |
| EF440434/East Timor/2000 | I | ✓ | ✓ | ✘ | ✓ | ✓ | ✓ |
| AY912458/Thailand/1998 | II | ✓ | ✓ | ✘ | ✓ | ✓ | ✓ |
| AY912455/Indonesia/1998 | I | ✓ | ✓ | ✘ | ✓ | ✓ | ✓ |
| DQ323042/India/2004 | III | ✓ | ✓ | ✓ | ✓ | ✓ | ✓ |
| EF629371/Brazil/2002 | V | ✓ | ✓ | ✘ | ✓ | ✓ | ✓ |
| EU052795/Argentina/2007 | III | ✓ | ✓ | ✘ | ✓ | ✓ | ✓ |
| EU052792/Argentina/2007 | III | ✓ | ✘ | ✘ | ✓ | ✘ | ✘ |
| AB189128/Indonesia/1998 | I | ✓ | ✓ | ✘ | ✓ | ✓ | ✓ |
| AB189127/Indonesia/1998 | I | ✓ | ✓ | ✘ | ✓ | ✓ | ✓ |
| AB189125/Indonesia/1998 | I | ✓ | ✓ | ✘ | ✓ | ✓ | ✓ |
| EU367962/China/2007 | II | ✓ | ✓ | ✘ | ✓ | ✓ | ✓ |
| GU370053/Singapore/2007 | III | ✓ | ✓ | ✘ | ✓ | ✓ | ✓ |
| GU370052/Singapore/2009 | I | ✓ | ✓ | ✘ | ✓ | ✓ | ✓ |
| GU363549/China/2009 | III | ✓ | ✓ | ✘ | ✓ | ✓ | ✓ |
| DQ863638/Thailand/1973 | II | ✓ | ✓ | ✘ | ✓ | ✓ | ✓ |
| AY770511/India/2003 | III | ✓ | ✓ | ✓ | ✓ | ✓ | ✓ |
| GQ466079/India/2008 | III | ✓ | ✘ | ✓ | ✓ | ✘ | ✘ |
| FJ644564/India/2007 | III | ✓ | ✘ | ✓ | ✓ | ✘ | ✘ |
| JF504679/China/2009 | III | ✓ | ✘ | ✘ | ✓ | ✘ | ✘ |
| HQ235027/Paraguay/2007 | III | ✓ | ✘ | ✘ | ✓ | ✘ | ✘ |
| JN697379/Brazil/2006 | V | ✓ | ✓ | ✘ | ✓ | ✓ | ✓ |
| JN662391/China/2009 | III | ✓ | ✘ | ✘ | ✓ | ✘ | ✘ |
| HQ332170/Venezuela/2006 | III | ✓ | ✓ | ✘ | ✓ | ✓ | ✓ |
| JQ411814/Sri Lanka/1989 | III | ✓ | ✓ | ✘ | ✓ | ✓ | ✓ |
| JQ339723/Pakistan/2011 | II | ✓ | ✓ | ✘ | ✓ | ✓ | ✓ |
| JQ045694/Vietnam/2011 | II | ✓ | ✓ | ✘ | ✓ | ✓ | ✓ |
| HQ332171/Venezuela/2006 | III | ✓ | ✘ | ✘ | ✓ | ✘ | ✘ |
| EU482461/Viet Nam/2007 | II | ✓ | ✓ | ✘ | ✓ | ✓ | ✓ |
| EU482460/Vietnam/2006 | II | ✓ | ✘ | ✘ | ✓ | ✘ | ✘ |
| EU529690/Venezuela/2001 | III | ✓ | ✘ | ✘ | ✓ | ✘ | ✘ |
| EU529683/Venezuela/2007 | III | ✓ | ✓ | ✘ | ✓ | ✓ | ✓ |
| EU482596/Puerto Rico/1998 | III | ✓ | ✘ | ✘ | ✓ | ✘ | ✘ |
| EU482595/Puerto Rico/2003 | III | ✓ | ✓ | ✘ | ✓ | ✓ | ✓ |
| EU482555/Puerto Rico/2006 | III | ✓ | ✓ | ✘ | ✓ | ✓ | ✓ |
| EU482462/Vietnam/2007 | II | ✓ | ✘ | ✘ | ✓ | ✘ | ✘ |
| EU660409/Vietnam/2006 | II | ✓ | ✓ | ✘ | ✓ | ✓ | ✓ |
| EU596494/USA/2007 | III | ✓ | ✓ | ✘ | ✓ | ✓ | ✓ |
| EU569691/Venezuela/2001 | III | ✓ | ✘ | ✘ | ✓ | ✘ | ✘ |
| EU529705/USA/2004 | III | ✓ | ✓ | ✘ | ✓ | ✓ | ✓ |
| EU529702/USA/2003 | III | ✓ | ✘ | ✘ | ✓ | ✘ | ✘ |
| EU529699/USA/2006 | III | ✓ | ✘ | ✘ | ✓ | ✘ | ✘ |
| EU529697/USA/2000 | III | ✓ | ✘ | ✘ | ✓ | ✘ | ✘ |
| EU529696/USA/1999 | III | ✓ | ✓ | ✘ | ✓ | ✓ | ✓ |
| EU687234/USA/2002 | III | ✓ | ✘ | ✘ | ✓ | ✘ | ✘ |
| EU660410/Vietnam/2006 | II | ✓ | ✘ | ✘ | ✓ | ✘ | ✘ |
| FJ182011/USA/2005 | III | ✓ | ✓ | ✘ | ✓ | ✓ | ✓ |
| FJ182006/USA/2004 | III | ✓ | ✘ | ✘ | ✓ | ✘ | ✘ |
| EU781137/USA/1999 | III | ✓ | ✘ | ✘ | ✓ | ✘ | ✘ |
| FJ024471/USA/2004 | III | ✓ | ✓ | ✘ | ✓ | ✓ | ✓ |
| EU854298/USA/2002 | III | ✓ | ✓ | ✘ | ✓ | ✓ | ✓ |
| FJ410177/USA/2000 | III | ✓ | ✘ | ✘ | ✓ | ✘ | ✘ |
| FJ373304/Venezuela/2004 | III | ✓ | ✓ | ✘ | ✓ | ✓ | ✓ |
| FJ547082/USA/2001 | III | ✓ | ✘ | ✘ | ✓ | ✘ | ✘ |
| FJ547066/Vietnam/2008 | II | ✓ | ✘ | ✘ | ✓ | ✘ | ✘ |
| FJ639757/Venezuela/2001 | III | ✓ | ✓ | ✘ | ✓ | ✓ | ✓ |
| FJ639750/Venezuela/2000 | III | ✓ | ✓ | ✘ | ✓ | ✓ | ✓ |
| FJ562103/Vietnam/2008 | II | ✓ | ✘ | ✘ | ✓ | ✘ | ✘ |
| FJ562102/Vietnam/2007 | II | ✓ | ✘ | ✘ | ✓ | ✘ | ✘ |
| FJ639779/Venezuela/2003 | III | ✓ | ✘ | ✘ | ✓ | ✘ | ✘ |
| FJ639778/Venezuela/2002 | III | ✓ | ✘ | ✘ | ✓ | ✘ | ✘ |
| FJ639805/Venezuela/2005 | III | ✓ | ✓ | ✘ | ✓ | ✓ | ✓ |
| FJ639801/Venezuela/2004 | III | ✓ | ✘ | ✘ | ✓ | ✘ | ✘ |
| FJ639786/Venezuela/2003 | III | ✓ | ✓ | ✘ | ✓ | ✓ | ✓ |
| FJ639713/Cambodia/2007 | II | ✓ | ✘ | ✘ | ✓ | ✘ | ✘ |
| FJ461338/Vietnam/2008 | II | ✓ | ✘ | ✘ | ✓ | ✘ | ✘ |
| FJ461329/Vietnam/2007 | II | ✓ | ✘ | ✘ | ✓ | ✘ | ✘ |
| FJ639826/Venezuela/2008 | III | ✓ | ✘ | ✘ | ✓ | ✘ | ✘ |
| FJ639825/Venezuela/2006 | III | ✓ | ✘ | ✘ | ✓ | ✘ | ✘ |
| FJ639816/Venezuela/2005 | III | ✓ | ✓ | ✘ | ✓ | ✓ | ✓ |
| FJ744727/Thailand/2001 | II | ✓ | ✓ | ✘ | ✓ | ✓ | ✓ |
| FJ744700/Venezuela/2001 | III | ✓ | ✘ | ✘ | ✓ | ✘ | ✘ |
| FJ639730/Cambodia/2006 | II | ✓ | ✓ | ✘ | ✓ | ✓ | ✓ |
| FJ639727/Cambodia/2005 | II | ✓ | ✘ | ✘ | ✓ | ✘ | ✘ |
| FJ639726/Cambodia/2004 | II | ✓ | ✘ | ✘ | ✓ | ✘ | ✘ |
| FJ639725/Cambodia/2003 | II | ✓ | ✓ | ✘ | ✓ | ✓ | ✓ |
| FJ639722/Cambodia/2002 | II | ✓ | ✓ | ✘ | ✓ | ✓ | ✓ |
| FJ639720/Cambodia/2001 | II | ✓ | ✓ | ✘ | ✓ | ✓ | ✓ |
| FJ639719/Cambodia/2000 | II | ✓ | ✓ | ✘ | ✓ | ✓ | ✓ |
| FJ639716/Cambodia/2008 | II | ✓ | ✘ | ✘ | ✓ | ✘ | ✘ |
| FJ639714/Cambodia/2007 | II | ✓ | ✓ | ✘ | ✓ | ✓ | ✓ |
| FJ850080/Brazil/2003 | III | ✓ | ✘ | ✘ | ✓ | ✘ | ✘ |
| FJ810414/Thailand/2001 | II | ✓ | ✘ | ✘ | ✓ | ✘ | ✘ |
| FJ882574/Sri Lanka/1985 | III | ✓ | ✓ | ✘ | ✓ | ✓ | ✓ |
| FJ882573/Sri Lanka/1993 | III | ✓ | ✓ | ✘ | ✓ | ✓ | ✓ |
| FJ882572/Sri Lanka/1989 | III | ✓ | ✓ | ✘ | ✓ | ✓ | ✓ |
| FJ873813/Nicaragua/2008 | III | ✓ | ✓ | ✘ | ✓ | ✓ | ✓ |
| FJ850111/Venezuela/2007 | III | ✓ | ✘ | ✘ | ✓ | ✘ | ✘ |
| FJ850094/Brazil/2008 | III | ✓ | ✓ | ✘ | ✓ | ✓ | ✓ |
| FJ850092/Brazil/2007 | III | ✓ | ✘ | ✘ | ✓ | ✘ | ✘ |
| FJ850089/Brazil/2006 | III | ✓ | ✘ | ✘ | ✓ | ✘ | ✘ |
| FJ850086/Brazil/2005 | III | ✓ | ✘ | ✘ | ✓ | ✘ | ✘ |
| FJ850083/Brazil/2004 | III | ✓ | ✘ | ✘ | ✓ | ✘ | ✘ |
| FJ898464/Guyana/2002 | III | ✓ | ✓ | ✘ | ✓ | ✓ | ✓ |
| FJ898463/Saint Lucia/2001 | III | ✓ | ✘ | ✘ | ✓ | ✘ | ✘ |
| FJ898462/Anguilla/2001 | III | ✓ | ✘ | ✘ | ✓ | ✘ | ✘ |
| FJ898459/Trinidad and Tobago/2002 | III | ✓ | ✓ | ✘ | ✓ | ✓ | ✓ |
| FJ898458/Peru/2002 | III | ✓ | ✘ | ✘ | ✓ | ✘ | ✘ |
| FJ898457/Ecuador/2000 | III | ✓ | ✘ | ✘ | ✓ | ✘ | ✘ |
| FJ898456/Samoa/1995 | I | ✓ | ✓ | ✘ | ✓ | ✓ | ✓ |
| FJ898455/Cook Islands/1991 | I | ✓ | ✘ | ✘ | ✓ | ✘ | ✘ |
| FJ898447/Brazil/2003 | III | ✓ | ✘ | ✘ | ✓ | ✘ | ✘ |
| FJ898446/Brazil/2001 | III | ✓ | ✘ | ✘ | ✓ | ✘ | ✘ |
| FJ898445/Colombia/2007 | III | ✓ | ✘ | ✘ | ✓ | ✘ | ✘ |
| FJ898444/Colombia/2005 | III | ✓ | ✓ | ✘ | ✓ | ✓ | ✓ |
| FJ898443/Colombia/2003 | III | ✓ | ✘ | ✘ | ✓ | ✘ | ✘ |
| FJ898442/Mexico/2007 | III | ✓ | ✓ | ✘ | ✓ | ✓ | ✓ |
| FJ898441/Mexico/2006 | III | ✓ | ✓ | ✘ | ✓ | ✓ | ✓ |
| FJ898440/Mexico/2003 | III | ✓ | ✘ | ✘ | ✓ | ✘ | ✘ |
| FJ882578/Venezuela/2001 | III | ✓ | ✘ | ✘ | ✓ | ✘ | ✘ |
| FJ882576/Nicaragua/1994 | III | ✓ | ✘ | ✘ | ✓ | ✘ | ✘ |
| FJ882575/Mozambique/1985 | III | ✓ | ✘ | ✘ | ✓ | ✘ | ✘ |
| GQ199887/Sri Lanka/1983 | III | ✓ | ✘ | ✘ | ✓ | ✘ | ✘ |
| GQ199886/Nicaragua/1998 | III | ✓ | ✓ | ✘ | ✓ | ✓ | ✓ |
| GQ199871/Nicaragua/2008 | III | ✓ | ✘ | ✘ | ✓ | ✘ | ✘ |
| GQ199865/Nicaragua/2009 | III | ✓ | ✓ | ✘ | ✓ | ✓ | ✓ |
| GQ868617/Trinidad and Tobago/2002 | III | ✓ | ✘ | ✘ | ✓ | ✘ | ✘ |
| GQ868616/Saint Lucia/2001 | III | ✓ | ✘ | ✘ | ✓ | ✘ | ✘ |
| GQ868634/Cambodia/2006 | II | ✓ | ✘ | ✘ | ✓ | ✘ | ✘ |
| GQ868629/Cambodia/2005 | II | ✓ | ✓ | ✘ | ✓ | ✓ | ✓ |
| GQ868627/Cambodia/2002 | II | ✓ | ✓ | ✘ | ✓ | ✓ | ✓ |
| GQ868626/Cambodia/2001 | II | ✓ | ✓ | ✘ | ✓ | ✓ | ✓ |
| GQ868593/Thailand/1973 | II | ✓ | ✘ | ✘ | ✓ | ✘ | ✘ |
| GQ868548/Brazil/2006 | III | ✓ | ✓ | ✘ | ✓ | ✓ | ✓ |
| GQ252674/Sri Lanka/1997 | III | ✓ | ✓ | ✘ | ✓ | ✓ | ✓ |
| GQ199891/Colombia/2001 | III | ✓ | ✘ | ✘ | ✓ | ✘ | ✘ |
| GQ199889/Sri Lanka/1983 | III | ✓ | ✘ | ✘ | ✓ | ✘ | ✘ |
| GU131855/Brazil/2006 | III | ✓ | ✓ | ✘ | ✓ | ✓ | ✓ |
| GQ868578/Colombia/2007 | III | ✓ | ✓ | ✘ | ✓ | ✓ | ✓ |
| GQ868577/Colombia/2005 | III | ✓ | ✘ | ✘ | ✓ | ✘ | ✘ |
| GQ868575/Colombia/2004 | III | ✓ | ✘ | ✘ | ✓ | ✘ | ✘ |
| GQ868574/Colombia/2003 | III | ✓ | ✘ | ✘ | ✓ | ✘ | ✘ |
| GQ868571/Colombia/2002 | III | ✓ | ✘ | ✘ | ✓ | ✘ | ✘ |
| GU131877/Brazil/2007 | III | ✓ | ✘ | ✘ | ✓ | ✘ | ✘ |
| GU131865/Brazil/2006 | III | ✓ | ✘ | ✘ | ✓ | ✘ | ✘ |
| GU131935/Cambodia/2007 | II | ✓ | ✘ | ✘ | ✓ | ✘ | ✘ |
| GU131933/Cambodia/2006 | II | ✓ | ✘ | ✘ | ✓ | ✘ | ✘ |
| GU131906/Cambodia/2003 | II | ✓ | ✘ | ✘ | ✓ | ✘ | ✘ |
| GU131905/Cambodia/2008 | II | ✓ | ✘ | ✘ | ✓ | ✘ | ✘ |
| GU131904/Cambodia/2005 | II | ✓ | ✓ | ✘ | ✓ | ✓ | ✓ |
| HM181972/Nicaragua/2009 | III | ✓ | ✓ | ✘ | ✓ | ✓ | ✓ |
| HM181935/Cambodia/2007 | II | ✓ | ✓ | ✘ | ✓ | ✓ | ✓ |
| HM181934/Cambodia/2006 | II | ✓ | ✘ | ✘ | ✓ | ✘ | ✘ |
| GU131950/Colombia/2001 | III | ✓ | ✘ | ✘ | ✓ | ✘ | ✘ |
| JF920394/Nicaragua/2009 | III | ✓ | ✘ | ✘ | ✓ | ✘ | ✘ |
| JF920397/Nicaragua/2010 | III | ✓ | ✓ | ✘ | ✓ | ✓ | ✓ |
| JN368477/Cambodia/2007 | II | ✓ | ✘ | ✘ | ✓ | ✘ | ✘ |
| JQ922555/India/1966 | III | ✓ | ✓ | ✓ | ✓ | ✓ | ✓ |
| JQ922554/USA/1963 | V | ✓ | ✘ | ✘ | ✓ | ✘ | ✘ |
| KC425219/Brazil/2002 | III | ✓ | ✓ | ✘ | ✓ | ✓ | ✓ |
| KC261634/China/2012 | II | ✓ | ✓ | ✘ | ✓ | ✓ | ✓ |
| JQ920489/Wallis and Futuna/1995 | I | ✓ | ✘ | ✘ | ✓ | ✘ | ✘ |
| JQ920486/New Caledonia/1996 | I | ✓ | ✘ | ✘ | ✓ | ✘ | ✘ |
| JQ920480/French Polynesia/1996 | I | ✓ | ✓ | ✘ | ✓ | ✓ | ✓ |
| JQ922557/India/2005 | III | ✓ | ✓ | ✓ | ✓ | ✓ | ✓ |
| JX669497/Brazil/2005 | III | ✓ | ✘ | ✘ | ✓ | ✘ | ✘ |
| JX669496/Brazil/2006 | III | ✓ | ✘ | ✘ | ✓ | ✘ | ✘ |
| JX669495/Brazil/2004 | III | ✓ | ✘ | ✘ | ✓ | ✘ | ✘ |
| JX669491/Brazil/2002 | III | ✓ | ✘ | ✘ | ✓ | ✘ | ✘ |
| JX669489/Brazil/2003 | III | ✓ | ✘ | ✘ | ✓ | ✘ | ✘ |
| JN406515/Australia/2008 | I | ✓ | ✓ | ✘ | ✓ | ✓ | ✓ |
| JN406514/Australia/1998 | II | ✓ | ✘ | ✘ | ✓ | ✘ | ✘ |
| JF808122/Paraguay/2003 | III | ✓ | ✘ | ✘ | ✓ | ✘ | ✘ |
| JF808121/Brazil/2007 | III | ✓ | ✓ | ✘ | ✓ | ✓ | ✓ |
| JF808120/Brazil/2009 | III | ✓ | ✘ | ✘ | ✓ | ✘ | ✘ |
| JF808119/Brazil/2004 | III | ✓ | ✘ | ✘ | ✓ | ✘ | ✘ |
| JF808118/Brazil/2002 | III | ✓ | ✘ | ✘ | ✓ | ✘ | ✘ |
| KF041259/Pakistan/2006 | III | ✓ | ✘ | ✘ | ✓ | ✘ | ✘ |
| KF041258/Pakistan/2009 | III | ✓ | ✘ | ✘ | ✓ | ✘ | ✘ |
| KF041255/Pakistan/2007 | III | ✓ | ✘ | ✘ | ✓ | ✘ | ✘ |
| KF041254/Pakistan/2008 | III | ✓ | ✓ | ✘ | ✓ | ✓ | ✓ |
| MN227703/China/2019 | III | ✓ | ✓ | ✘ | ✓ | ✓ | ✓ |
| MN227702/China/2019 | III | ✓ | ✘ | ✘ | ✓ | ✘ | ✘ |
| MN227701/China/2019 | III | ✓ | ✘ | ✘ | ✓ | ✘ | ✘ |
| MN227700/China/2019 | III | ✓ | ✘ | ✘ | ✓ | ✘ | ✘ |
| MN227699/China/2019 | III | ✓ | ✘ | ✘ | ✓ | ✘ | ✘ |
| MN227698/China/2019 | III | ✓ | ✘ | ✘ | ✓ | ✘ | ✘ |
| MN227697/Maldives/2019 | III | ✓ | ✘ | ✘ | ✓ | ✘ | ✘ |
| EF428575/Brazil/2003 | V | ✓ | ✓ | ✘ | ✓ | ✓ | ✓ |
| KU216209/India/2013 | III | ✓ | ✓ | ✓ | ✓ | ✓ | ✓ |
| KJ737430/Thailand/1983 | II | ✓ | ✘ | ✘ | ✓ | ✘ | ✘ |
| KJ737429/Thailand/1994 | II | ✓ | ✘ | ✘ | ✓ | ✘ | ✘ |
| KF954949/China/2013 | III | ✓ | ✘ | ✘ | ✓ | ✘ | ✘ |
| GU189648/China/2009 | II | ✓ | ✘ | ✘ | ✓ | ✘ | ✘ |
| MW426463/China/2014 | II | ✓ | ✓ | ✘ | ✓ | ✓ | ✓ |
| MW308181/Mexico/2019 | III | ✓ | ✓ | ✘ | ✓ | ✓ | ✓ |
| MW288040/Senegal/2018 | III | ✓ | ✓ | ✘ | ✓ | ✓ | ✓ |
| MN448993/Thailand/2012 | II | ✓ | ✓ | ✘ | ✓ | ✓ | ✓ |
| MN448989/Thailand/2011 | II | ✓ | ✓ | ✘ | ✓ | ✓ | ✓ |
| MN253131/India/2018 | III | ✓ | ✓ | ✓ | ✓ | ✓ | ✓ |
| MT949456/India/2019 | III | ✓ | ✓ | ✓ | ✓ | ✓ | ✓ |
| MT921575/Australia/2016 | I | ✓ | ✓ | ✘ | ✓ | ✓ | ✓ |
| MT921574/Australia/2017 | I | ✓ | ✓ | ✘ | ✓ | ✓ | ✓ |
| MT006158/Sri Lanka/2018 | I | ✓ | ✓ | ✘ | ✓ | ✓ | ✓ |
| MT076948/Kenya/2015 | V | ✓ | ✓ | ✘ | ✓ | ✓ | ✓ |
| LC436677/Bangladesh/2017 | I | ✓ | ✓ | ✘ | ✓ | ✓ | ✓ |
| LC379197/Gabon/2017 | III | ✓ | ✓ | ✘ | ✓ | ✓ | ✓ |
| MT261979/Burkina Faso/2017 | III | ✓ | ✘ | ✘ | ✓ | ✘ | ✘ |
| MH051732/Malaysia/2014 | I | ✓ | ✓ | ✘ | ✓ | ✓ | ✓ |
| MT224921/India/2014 | III | ✓ | ✘ | ✓ | ✓ | ✘ | ✘ |
| MT224920/India/2014 | III | ✓ | ✓ | ✓ | ✓ | ✓ | ✓ |
| MN964274/China/2019 | III | ✓ | ✘ | ✘ | ✓ | ✘ | ✘ |
| MN018389/China/2017 | I | ✓ | ✓ | ✘ | ✓ | ✓ | ✓ |
| MN018385/India/2016 | III | ✓ | ✘ | ✓ | ✓ | ✘ | ✘ |
| MN018371/China/2015 | III | ✓ | ✘ | ✘ | ✓ | ✘ | ✘ |
| MN018370/China/2015 | I | ✓ | ✓ | ✘ | ✓ | ✓ | ✓ |
| MN922041/China/2019 | I | ✓ | ✓ | ✘ | ✓ | ✓ | ✓ |
| MK614073/Colombia/2014 | III | ✓ | ✓ | ✘ | ✓ | ✓ | ✓ |
| MH891766/India/2017 | III | ✓ | ✓ | ✓ | ✓ | ✓ | ✓ |
| MK005258/Malaysia/2014 | I | ✓ | ✓ | ✘ | ✓ | ✓ | ✓ |
| MK829116/India/2018 | III | ✓ | ✓ | ✓ | ✓ | ✓ | ✓ |
| MN453624/Singapore/2016 | III | ✓ | ✘ | ✘ | ✓ | ✘ | ✘ |
| MK002764/India/2017 | III | ✓ | ✘ | ✓ | ✓ | ✘ | ✘ |
| MN365225/India/2018 | III | ✓ | ✓ | ✓ | ✓ | ✓ | ✓ |
| MN124077/Brazil/2003 | III | ✓ | ✓ | ✘ | ✓ | ✓ | ✓ |
| MN124076/Brazil/2003 | III | ✓ | ✓ | ✘ | ✓ | ✓ | ✓ |
| MK894341/China/2018 | III | ✓ | ✓ | ✘ | ✓ | ✓ | ✓ |
| MK894338/Thailand/2018 | I | ✓ | ✓ | ✘ | ✓ | ✓ | ✓ |
| MK796419/India/2018 | III | ✓ | ✓ | ✓ | ✓ | ✓ | ✓ |
| MK796412/India/2016 | III | ✓ | ✓ | ✓ | ✓ | ✓ | ✓ |
| MH708493/Russia/2014 | II | ✓ | ✓ | ✘ | ✓ | ✓ | ✓ |
| MH734372/India/2007 | III | ✓ | ✓ | ✓ | ✓ | ✓ | ✓ |
| MK506265/Thailand/2007 | V | ✓ | ✘ | ✘ | ✓ | ✘ | ✘ |
| MH823209/Indonesia/2016 | I | ✓ | ✓ | ✘ | ✓ | ✓ | ✓ |
| MG932069/Malaysia/2017 | III | ✓ | ✓ | ✘ | ✓ | ✓ | ✓ |
| MH822957/India/2013 | III | ✓ | ✘ | ✓ | ✓ | ✘ | ✘ |
| LC410195/Thailand/2017 | III | ✓ | ✘ | ✘ | ✓ | ✘ | ✘ |
| MF682975/China/2013 | II | ✓ | ✘ | ✘ | ✓ | ✘ | ✘ |
| MH544651/Colombia/2016 | III | ✓ | ✘ | ✘ | ✓ | ✘ | ✘ |
| MH544650/Colombia/2007 | III | ✓ | ✓ | ✘ | ✓ | ✓ | ✓ |
| KY794790/Papua New Guinea/2008 | I | ✓ | ✓ | ✘ | ✓ | ✓ | ✓ |
| KY849775/Laos/2010 | II | ✓ | ✓ | ✘ | ✓ | ✓ | ✓ |
| KY234197/Thailand/2012 | V | ✓ | ✘ | ✘ | ✓ | ✘ | ✘ |
| MF142763/Thailand/2015 | III | ✓ | ✘ | ✘ | ✓ | ✘ | ✘ |
| MF370226/China/2013 | III | ✓ | ✘ | ✘ | ✓ | ✘ | ✘ |
| KY921907/Singapore/2015 | III | ✓ | ✘ | ✘ | ✓ | ✘ | ✘ |
| AB214882/Timor Leste/2005 | I | ✓ | ✘ | ✘ | ✓ | ✘ | ✘ |
| KU509286/India/2011 | III | ✓ | ✓ | ✓ | ✓ | ✓ | ✓ |
| KT424097/Thailand/2014 | II | ✓ | ✓ | ✘ | ✓ | ✓ | ✓ |
| KX518580/Sri Lanka/2004 | III | ✓ | ✘ | ✘ | ✓ | ✘ | ✘ |
| HM162761/Brazil/2010 | V | ✓ | ✘ | ✘ | ✓ | ✘ | ✘ |
| AY858048/Indonesia/2004 | I | ✓ | ✓ | ✘ | ✓ | ✓ | ✓ |
| KX380842/Singapore/2013 | III | ✓ | ✘ | ✘ | ✓ | ✘ | ✘ |
| DQ863637/Thailand/2000 | II | ✓ | ✓ | ✘ | ✓ | ✓ | ✓ |
| DQ863622/Thailand/1996 | II | ✓ | ✓ | ✘ | ✓ | ✓ | ✓ |
| DQ863613/Thailand/1990 | II | ✓ | ✘ | ✘ | ✓ | ✘ | ✘ |
| DQ863594/Thailand/1987 | II | ✓ | ✓ | ✘ | ✓ | ✓ | ✓ |
| DQ863570/Thailand/1981 | II | ✓ | ✓ | ✘ | ✓ | ✓ | ✓ |
| KT726361/Cuba/2002 | III | ✓ | ✓ | ✘ | ✓ | ✓ | ✓ |
| KT726360/Cuba/2001 | III | ✓ | ✘ | ✘ | ✓ | ✘ | ✘ |
| KU050695/Philippines/1956 | V | ✓ | ✘ | ✘ | ✓ | ✘ | ✘ |
| KP406805/South Korea/2015 | V | ✓ | ✘ | ✘ | ✓ | ✘ | ✘ |
| KM217136/Pakistan/2011 | III | ✓ | ✓ | ✘ | ✓ | ✓ | ✓ |
| KR296744/China/2013 | II | ✓ | ✘ | ✘ | ✓ | ✘ | ✘ |
| KC762693/Indonesia/2010 | I | ✓ | ✓ | ✘ | ✓ | ✓ | ✓ |
| KC762691/Indonesia/2008 | I | ✓ | ✓ | ✘ | ✓ | ✓ | ✓ |
| KC762684/Indonesia/2007 | I | ✓ | ✓ | ✘ | ✓ | ✓ | ✓ |
| KR087027/India/2014 | III | ✓ | ✓ | ✓ | ✓ | ✓ | ✓ |
| KM190937/Philippines/1964 | V | ✓ | ✘ | ✘ | ✓ | ✘ | ✘ |
| KJ622199/China/2013 | II | ✓ | ✓ | ✘ | ✓ | ✓ | ✓ |
| KF973487/Nicaragua/2011 | III | ✓ | ✘ | ✘ | ✓ | ✘ | ✘ |
| KF973480/Nicaragua/2012 | III | ✓ | ✓ | ✘ | ✓ | ✓ | ✓ |
| KF971711/Nicaragua/2010 | III | ✓ | ✘ | ✘ | ✓ | ✘ | ✘ |
| KF971706/Nicaragua/2009 | III | ✓ | ✘ | ✘ | ✓ | ✘ | ✘ |
| KJ830751/Saudi Arabia/2014 | III | ✓ | ✓ | ✘ | ✓ | ✓ | ✓ |
| KJ643590/Peru/2007 | III | ✓ | ✓ | ✘ | ✓ | ✓ | ✓ |
| KJ189291/Peru/2008 | III | ✓ | ✘ | ✘ | ✓ | ✘ | ✘ |
| KJ189293/Peru/2005 | III | ✓ | ✘ | ✘ | ✓ | ✘ | ✘ |
| KJ189290/Peru/2008 | III | ✓ | ✘ | ✘ | ✓ | ✘ | ✘ |
| KJ189283/Peru/2007 | III | ✓ | ✘ | ✘ | ✓ | ✘ | ✘ |
| KJ189266/Peru/2004 | III | ✓ | ✓ | ✘ | ✓ | ✓ | ✓ |
| KJ189261/Peru/2008 | III | ✓ | ✘ | ✘ | ✓ | ✘ | ✘ |
| KJ189259/Peru/2002 | III | ✓ | ✘ | ✘ | ✓ | ✘ | ✘ |
| KF824903/China/2013 | II | ✓ | ✘ | ✘ | ✓ | ✘ | ✘ |
| KF543368/India/2011 | III | ✓ | ✓ | ✓ | ✓ | ✓ | ✓ |
| KF955507/Cambodia/2007 | II | ✓ | ✘ | ✘ | ✓ | ✘ | ✘ |
| KF955505/Grenada/2002 | III | ✓ | ✘ | ✘ | ✓ | ✘ | ✘ |
| KF955491/Nicaragua/2008 | III | ✓ | ✘ | ✘ | ✓ | ✘ | ✘ |
| KF955487/Venezuela/2001 | III | ✓ | ✓ | ✘ | ✓ | ✓ | ✓ |
| KF955480/Venezuela/2001 | III | ✓ | ✓ | ✘ | ✓ | ✓ | ✓ |
| KF955477/India/1984 | II | ✓ | ✘ | ✓ | ✓ | ✘ | ✘ |
| KF955476/Sri Lanka/1983 | II | ✓ | ✘ | ✘ | ✓ | ✘ | ✘ |
| KF955474/Sri Lanka/1989 | III | ✓ | ✘ | ✘ | ✓ | ✘ | ✘ |
| KF955473/Brazil/2002 | III | ✓ | ✘ | ✘ | ✓ | ✘ | ✘ |
| KF955472/Venezuela/2004 | III | ✓ | ✘ | ✘ | ✓ | ✘ | ✘ |
| KF955468/Puerto Rico/2001 | III | ✓ | ✘ | ✘ | ✓ | ✘ | ✘ |
| KF955466/Puerto Rico/2000 | III | ✓ | ✘ | ✘ | ✓ | ✘ | ✘ |
| KF955464/Cambodia/2007 | II | ✓ | ✘ | ✘ | ✓ | ✘ | ✘ |
| KF955463/Cambodia/2001 | II | ✓ | ✓ | ✘ | ✓ | ✓ | ✓ |
| KF955461/Cambodia/1999 | II | ✓ | ✓ | ✘ | ✓ | ✓ | ✓ |
| KF955460/Vietnam/2008 | II | ✓ | ✘ | ✘ | ✓ | ✘ | ✘ |
| KF955458/Vietnam/2007 | II | ✓ | ✘ | ✘ | ✓ | ✘ | ✘ |
| KF955456/Puerto Rico/2006 | III | ✓ | ✘ | ✘ | ✓ | ✘ | ✘ |
| KF955453/Venezuela/2013 | III | ✓ | ✘ | ✘ | ✓ | ✘ | ✘ |
| KF955336/Nicaragua/2008 | III | ✓ | ✘ | ✘ | ✓ | ✘ | ✘ |
| KF955335/Nicaragua/2009 | III | ✓ | ✘ | ✘ | ✓ | ✘ | ✘ |
| KF955333/Cambodia/2005 | II | ✓ | ✘ | ✘ | ✓ | ✘ | ✘ |
| KF955332/Cambodia/2000 | II | ✓ | ✘ | ✘ | ✓ | ✘ | ✘ |
| KF921928/Nicaragua/2010 | III | ✓ | ✘ | ✘ | ✓ | ✘ | ✘ |
| JF808129/Paraguay/2003 | III | ✓ | ✘ | ✘ | ✓ | ✘ | ✘ |
| JF808127/Brazil/2002 | III | ✓ | ✘ | ✘ | ✓ | ✘ | ✘ |
| JF808126/Brazil/2003 | III | ✓ | ✘ | ✘ | ✓ | ✘ | ✘ |
| MH544650/Colombia/2015 | III | ✓ | ✘ | ✘ | ✓ | ✘ | ✘ |
| MF004386/Malaysia/2012 | I | ✓ | ✓ | ✘ | ✓ | ✓ | ✓ |
| KY670634/Taiwan/1998 | II | ✓ | ✓ | ✘ | ✓ | ✓ | ✓ |
| LT898452/Malaysia/2011 | III | ✓ | ✓ | ✘ | ✓ | ✓ | ✓ |
| KX518573/Sri Lanka/1989 | III | ✓ | ✘ | ✘ | ✓ | ✘ | ✘ |
| EF428573/Brazil/2002 | V | ✓ | ✓ | ✘ | ✓ | ✓ | ✓ |
| MN448983/Thailand/2011 | II | ✓ | ✓ | ✘ | ✓ | ✓ | ✓ |
| LT996912/Malaysia/1987 | III | ✓ | ✘ | ✘ | ✓ | ✘ | ✘ |
| MK858155/India/2017 | III | ✓ | ✓ | ✓ | ✓ | ✓ | ✓ |
| MT406776/India/2019 | III | ✓ | ✓ | ✓ | ✓ | ✓ | ✓ |
| MN083246/Sri Lanka/2017 | I | ✓ | ✘ | ✘ | ✓ | ✘ | ✘ |
| MK829114/India/2018 | III | ✓ | ✘ | ✓ | ✓ | ✘ | ✘ |
| MH888333/Bolivia/2011 | III | ✓ | ✘ | ✘ | ✓ | ✘ | ✘ |
| MH888332/Thailand/2012 | II | ✓ | ✘ | ✘ | ✓ | ✘ | ✘ |
| MF682973/China/2013 | II | ✓ | ✘ | ✘ | ✓ | ✘ | ✘ |
| MF682970/China/2016 | I | ✓ | ✘ | ✘ | ✓ | ✘ | ✘ |
| AH011667/Martinique/2001 | III | ✓ | ✘ | ✘ | ✓ | ✘ | ✘ |
| MW295815/Myanmar/2020 | I | ✓ | ✓ | ✘ | ✓ | ✓ | ✓ |

**c. DENV-4**

| **Sequence detail**  **Gen Bank Accession no./Country**  **/Year of collection** | **Genotype** | **Phylogenetic analysis**  **(n=186)** | **Molecular clock**  **(n=134)** | **BSP**  **(n=23)** | **Network**  **(n=186)** | **Selection pressure**  **(n=134)** | **Entropy**  **(n=134)** |
| --- | --- | --- | --- | --- | --- | --- | --- |
| KR011349/Philippines/1956 | I | ✓ | ✓ | ✘ | ✓ | ✓ | ✓ |
| OK173641/India/2018 | I | ✓ | ✓ | ✓ | ✓ | ✓ | ✓ |
| OK173642/India/2018 | I | ✓ | ✓ | ✓ | ✓ | ✓ | ✓ |
| KY924607/Viet Nam/2016 | I | ✓ | ✓ | ✘ | ✓ | ✓ | ✓ |
| JX024758/Singapore/2010 | VI | ✓ | ✓ | ✘ | ✓ | ✓ | ✓ |
| JX024757/Singapore/2010 | VI | ✓ | ✘ | ✘ | ✓ | ✘ | ✘ |
| MW793460/Thailand/2021 | II | ✓ | ✓ | ✘ | ✓ | ✓ | ✓ |
| MW793459/Thailand/2021 | II | ✓ | ✘ | ✘ | ✓ | ✘ | ✘ |
| MT040677/Paraguay/2018 | II | ✓ | ✓ | ✘ | ✓ | ✓ | ✓ |
| MW301595/Malaysia/2020 | VI | ✓ | ✓ | ✘ | ✓ | ✓ | ✓ |
| MW295825/Myanmar/2017 | I | ✓ | ✓ | ✘ | ✓ | ✓ | ✓ |
| MN449002/Thailand/2011 | I | ✓ | ✓ | ✘ | ✓ | ✓ | ✓ |
| MN448998/Thailand/2012 | I | ✓ | ✘ | ✘ | ✓ | ✘ | ✘ |
| MN448997/Thailand/2011 | I | ✓ | ✓ | ✘ | ✓ | ✓ | ✓ |
| MG973744/India/2017 | I | ✓ | ✓ | ✓ | ✓ | ✓ | ✓ |
| MG973742/India/2017 | I | ✓ | ✓ | ✓ | ✓ | ✓ | ✓ |
| MN239489/India/2017 | I | ✓ | ✓ | ✓ | ✓ | ✓ | ✓ |
| MT076955/Kenya/2015 | II | ✓ | ✘ | ✘ | ✓ | ✘ | ✘ |
| MK858146/India/2016 | I | ✓ | ✓ | ✓ | ✓ | ✓ | ✓ |
| MK858143/India/2019 | I | ✓ | ✓ | ✓ | ✓ | ✓ | ✓ |
| MN192436/USA/2016 | II | ✓ | ✓ | ✘ | ✓ | ✓ | ✓ |
| MH051734/Malaysia/2014 | VI | ✓ | ✓ | ✘ | ✓ | ✓ | ✓ |
| MT182025/India/2017 | I | ✓ | ✓ | ✓ | ✓ | ✓ | ✓ |
| MT182024/India/2017 | I | ✓ | ✓ | ✓ | ✓ | ✓ | ✓ |
| MN018398/China/2016 | I | ✓ | ✓ | ✘ | ✓ | ✓ | ✓ |
| MN018396/Thailand/2015 | VI | ✓ | ✓ | ✘ | ✓ | ✓ | ✓ |
| KJ579245/Brazil/2012 | II | ✓ | ✓ | ✘ | ✓ | ✓ | ✓ |
| KJ596658/Brazil/2012 | II | ✓ | ✓ | ✘ | ✓ | ✓ | ✓ |
| KJ596660/Brazil/2012 | II | ✓ | ✘ | ✘ | ✓ | ✘ | ✘ |
| KJ596665/Brazil/2012 | II | ✓ | ✘ | ✘ | ✓ | ✘ | ✘ |
| KJ596671/Brazil/2012 | II | ✓ | ✘ | ✘ | ✓ | ✘ | ✘ |
| MH891769/India/2017 | I | ✓ | ✓ | ✓ | ✓ | ✓ | ✓ |
| MK829120/India/2018 | I | ✓ | ✓ | ✓ | ✓ | ✓ | ✓ |
| MK796413/India/2016 | I | ✓ | ✓ | ✓ | ✓ | ✓ | ✓ |
| MK789303/India/2017 | I | ✓ | ✓ | ✓ | ✓ | ✓ | ✓ |
| MK640208/China/2018 | VI | ✓ | ✓ | ✘ | ✓ | ✓ | ✓ |
| MK614093/China/2018 | I | ✓ | ✓ | ✘ | ✓ | ✓ | ✓ |
| MK614092/China/2018 | I | ✓ | ✓ | ✘ | ✓ | ✓ | ✓ |
| MK614088/China/2019 | I | ✓ | ✓ | ✘ | ✓ | ✓ | ✓ |
| MK514144/Haiti/2015 | II | ✓ | ✘ | ✘ | ✓ | ✘ | ✘ |
| MK506266/Thailand/2007 | I | ✓ | ✓ | ✘ | ✓ | ✓ | ✓ |
| KY672960/China/2015 | I | ✓ | ✓ | ✘ | ✓ | ✓ | ✓ |
| KY672956/China/2015 | I | ✓ | ✓ | ✘ | ✓ | ✓ | ✓ |
| MH823210/Indonesia/2014 | VI | ✓ | ✓ | ✘ | ✓ | ✓ | ✓ |
| MH888334/Malaysia/2013 | VI | ✓ | ✓ | ✘ | ✓ | ✓ | ✓ |
| LC410203/Thailand/2017 | I | ✓ | ✓ | ✘ | ✓ | ✓ | ✓ |
| LC410198/Thailand/2016 | I | ✓ | ✓ | ✘ | ✓ | ✓ | ✓ |
| MH382789/New Guinea/2016 | VI | ✓ | ✓ | ✘ | ✓ | ✓ | ✓ |
| MG182049/Malaysia/2016 | VI | ✓ | ✓ | ✘ | ✓ | ✓ | ✓ |
| MG182046/Malaysia/2016 | VI | ✓ | ✘ | ✘ | ✓ | ✘ | ✘ |
| MF004387/Senegal/1981 | II | ✓ | ✓ | ✘ | ✓ | ✓ | ✓ |
| MG272274/India/2016 | I | ✓ | ✓ | ✓ | ✓ | ✓ | ✓ |
| MG053170/India/2016 | I | ✓ | ✓ | ✓ | ✓ | ✓ | ✓ |
| MG053167/India/2016 | I | ✓ | ✓ | ✓ | ✓ | ✓ | ✓ |
| MG053162/India/2016 | I | ✓ | ✓ | ✓ | ✓ | ✓ | ✓ |
| MG601754/China/2013 | I | ✓ | ✓ | ✘ | ✓ | ✓ | ✓ |
| KY849762/Laos/2009 | I | ✓ | ✓ | ✘ | ✓ | ✓ | ✓ |
| KY670635/Taiwan/2003 | I | ✓ | ✓ | ✘ | ✓ | ✓ | ✓ |
| KY084521/Brazil/2013 | II | ✓ | ✓ | ✘ | ✓ | ✓ | ✓ |
| KY084515/Brazil/2012 | II | ✓ | ✓ | ✘ | ✓ | ✓ | ✓ |
| KY084511/Brazil/2011 | II | ✓ | ✓ | ✘ | ✓ | ✓ | ✓ |
| KY921910/Singapore/2016 | VI | ✓ | ✓ | ✘ | ✓ | ✓ | ✓ |
| KX224312/Singapore/2014 | I | ✓ | ✓ | ✘ | ✓ | ✓ | ✓ |
| KX845005/India/2015 | I | ✓ | ✓ | ✓ | ✓ | ✓ | ✓ |
| KP792537/Singapore/2011 | I | ✓ | ✓ | ✘ | ✓ | ✓ | ✓ |
| KU509288/Indonesia/2010 | VI | ✓ | ✓ | ✘ | ✓ | ✓ | ✓ |
| KU509287/India/2009 | I | ✓ | ✓ | ✓ | ✓ | ✓ | ✓ |
| KU523872/Indonesia/2015 | VI | ✓ | ✓ | ✘ | ✓ | ✓ | ✓ |
| KU523871/Philippines/2014 | VI | ✓ | ✓ | ✘ | ✓ | ✓ | ✓ |
| FJ439174/Philippines/1956 | I | ✓ | ✘ | ✘ | ✓ | ✘ | ✘ |
| HQ875339/Singapore/2008 | VI | ✓ | ✓ | ✘ | ✓ | ✓ | ✓ |
| HQ840706/Brazil/2009 | I | ✓ | ✓ | ✘ | ✓ | ✓ | ✓ |
| AY858050/Indonesia/2004 | VI | ✓ | ✓ | ✘ | ✓ | ✓ | ✓ |
| KP188566/Brazil/2013 | II | ✓ | ✘ | ✘ | ✓ | ✘ | ✘ |
| KP188562/Brazil/2012 | II | ✓ | ✘ | ✘ | ✓ | ✘ | ✘ |
| KP188564/Brazil/2013 | II | ✓ | ✘ | ✘ | ✓ | ✘ | ✘ |
| KP188560/Brazil/2012 | II | ✓ | ✘ | ✘ | ✓ | ✘ | ✘ |
| KP188557/Brazil/2012 | II | ✓ | ✘ | ✘ | ✓ | ✘ | ✘ |
| KU513441/Brazil/2013 | II | ✓ | ✘ | ✘ | ✓ | ✘ | ✘ |
| AH012032/Dominica/1981 | II | ✓ | ✘ | ✘ | ✓ | ✘ | ✘ |
| AH012031/Puerto Rico/1982 | II | ✓ | ✘ | ✘ | ✓ | ✘ | ✘ |
| AH012023./Puerto Rico/1982 | II | ✓ | ✘ | ✘ | ✓ | ✘ | ✘ |
| AH012017/El Salvador/1993 | II | ✓ | ✓ | ✘ | ✓ | ✓ | ✓ |
| AH012015/Ecuador/1994 | II | ✓ | ✓ | ✘ | ✓ | ✓ | ✓ |
| AH012014/Puerto Rico/1994 | II | ✓ | ✓ | ✘ | ✓ | ✓ | ✓ |
| AH012013/Puerto Rico/1987 | II | ✓ | ✘ | ✘ | ✓ | ✘ | ✘ |
| AH012012/Puerto Rico/1986 | II | ✓ | ✘ | ✘ | ✓ | ✘ | ✘ |
| AH011995/Puerto Rico/1992 | II | ✓ | ✓ | ✘ | ✓ | ✓ | ✓ |
| AH011968/Costa Rica/1996 | II | ✓ | ✓ | ✘ | ✓ | ✓ | ✓ |
| AH011967/Martinique/1995 | II | ✓ | ✓ | ✘ | ✓ | ✓ | ✓ |
| AH011964/Puerto Rico/1998 | II | ✓ | ✓ | ✘ | ✓ | ✓ | ✓ |
| KP140942/Haiti/2014 | II | ✓ | ✓ | ✘ | ✓ | ✓ | ✓ |
| KT794007/Brazil/2011 | II | ✓ | ✘ | ✘ | ✓ | ✘ | ✘ |
| LC069810/Japan/2015 | VI | ✓ | ✓ | ✘ | ✓ | ✓ | ✓ |
| KJ534635/Costa Rica/2012 | II | ✓ | ✓ | ✘ | ✓ | ✓ | ✓ |
| KP406806/South Korea/2015 | I | ✓ | ✘ | ✘ | ✓ | ✘ | ✘ |
| KT261281/Brazil/2012 | II | ✓ | ✓ | ✘ | ✓ | ✓ | ✓ |
| KT261280/Brazil/2013 | II | ✓ | ✘ | ✘ | ✓ | ✘ | ✘ |
| KR922405/Thailand/2011 | I | ✓ | ✓ | ✘ | ✓ | ✓ | ✓ |
| KT276273/Haiti/2014 | II | ✓ | ✘ | ✘ | ✓ | ✘ | ✘ |
| KP723482/China/2010 | VI | ✓ | ✓ | ✘ | ✓ | ✓ | ✓ |
| KC762699/Indonesia/2008 | VI | ✓ | ✓ | ✘ | ✓ | ✓ | ✓ |
| KC762698/Indonesia/2008 | VI | ✓ | ✓ | ✘ | ✓ | ✓ | ✓ |
| KC762697/Indonesia/2007 | VI | ✓ | ✓ | ✘ | ✓ | ✓ | ✓ |
| KM190936/Thailand/2006 | II | ✓ | ✓ | ✘ | ✓ | ✓ | ✓ |
| KF286635/Brazil/2013 | II | ✓ | ✓ | ✘ | ✓ | ✓ | ✓ |
| KF286634/Brazil/2013 | II | ✓ | ✘ | ✘ | ✓ | ✘ | ✘ |
| KF955534/Thailand/2007 | I | ✓ | ✓ | ✘ | ✓ | ✓ | ✓ |
| KF955533/Thailand/2006 | I | ✓ | ✓ | ✘ | ✓ | ✓ | ✓ |
| KF955522/Thailand/2006 | I | ✓ | ✓ | ✘ | ✓ | ✓ | ✓ |
| KF955520/Thailand/2005 | I | ✓ | ✘ | ✘ | ✓ | ✘ | ✘ |
| KF955515/Cambodia/2007 | I | ✓ | ✓ | ✘ | ✓ | ✓ | ✓ |
| KF955514/Cambodia/2001 | I | ✓ | ✘ | ✘ | ✓ | ✘ | ✘ |
| KF955513/Cambodia/2008 | I | ✓ | ✘ | ✘ | ✓ | ✘ | ✘ |
| KF955510/Cambodia/2002 | I | ✓ | ✓ | ✘ | ✓ | ✓ | ✓ |
| KF041260/Pakistan/2009 | I | ✓ | ✓ | ✘ | ✓ | ✓ | ✓ |
| JQ247980/Brazil/2009 | II | ✓ | ✓ | ✘ | ✓ | ✓ | ✓ |
| KF543273/Cambodia/2011 | I | ✓ | ✓ | ✘ | ✓ | ✓ | ✓ |
| KF543272/Cambodia/2010 | I | ✓ | ✓ | ✘ | ✓ | ✓ | ✓ |
| KC333651/China/2012 | VI | ✓ | ✓ | ✘ | ✓ | ✓ | ✓ |
| JN638572/Cambodia/2008 | I | ✓ | ✘ | ✘ | ✓ | ✘ | ✘ |
| JN638571/Cambodia/2007 | I | ✓ | ✘ | ✘ | ✓ | ✘ | ✘ |
| JN638570/Cambodia/2008 | I | ✓ | ✘ | ✘ | ✓ | ✘ | ✘ |
| JQ915090/Wallis and Futuna/2009 | VI | ✓ | ✓ | ✘ | ✓ | ✓ | ✓ |
| JQ915089/New Caledonia/2009 | VI | ✓ | ✓ | ✘ | ✓ | ✓ | ✓ |
| JQ915088/New Caledonia/2009 | VI | ✓ | ✘ | ✘ | ✓ | ✘ | ✘ |
| JQ915085/New Caledonia/2008 | VI | ✓ | ✓ | ✘ | ✓ | ✓ | ✓ |
| JQ915084/French Polynesia/2010 | VI | ✓ | ✓ | ✘ | ✓ | ✓ | ✓ |
| JQ915083/French Polynesia/2009 | VI | ✓ | ✓ | ✘ | ✓ | ✓ | ✓ |
| JQ922560/India/2009 | I | ✓ | ✓ | ✓ | ✓ | ✓ | ✓ |
| JQ922559/India/1979 | I | ✓ | ✓ | ✓ | ✓ | ✓ | ✓ |
| JQ922558/India/1962 | V | ✓ | ✓ | ✓ | ✓ | ✓ | ✓ |
| JQ513345/Brazil/2011 | I | ✓ | ✓ | ✘ | ✓ | ✓ | ✓ |
| JQ513344/Brazil/2011 | II | ✓ | ✓ | ✘ | ✓ | ✓ | ✓ |
| JQ513341/Brazil/2010 | II | ✓ | ✓ | ✘ | ✓ | ✓ | ✓ |
| JQ513334/Brazil/2010 | II | ✓ | ✓ | ✘ | ✓ | ✓ | ✓ |
| JQ045566/USA/2010 | II | ✓ | ✓ | ✘ | ✓ | ✓ | ✓ |
| JN559741/Brazil/2010 | II | ✓ | ✘ | ✘ | ✓ | ✘ | ✘ |
| JN559740/Brazil/1982 | II | ✓ | ✘ | ✘ | ✓ | ✘ | ✘ |
| JN819409/Venezuela/1998 | II | ✓ | ✓ | ✘ | ✓ | ✓ | ✓ |
| JN819406/Venezuela/2006 | II | ✓ | ✓ | ✘ | ✓ | ✓ | ✓ |
| GQ868585/Colombia/2005 | II | ✓ | ✘ | ✘ | ✓ | ✘ | ✘ |
| GQ868584/Colombia/2004 | II | ✓ | ✓ | ✘ | ✓ | ✓ | ✓ |
| GQ868582/Colombia/2001 | II | ✓ | ✓ | ✘ | ✓ | ✓ | ✓ |
| GQ868645/Venezuela/2007 | II | ✓ | ✘ | ✘ | ✓ | ✘ | ✘ |
| GQ868594/Philippines/1956 | I | ✓ | ✘ | ✘ | ✓ | ✘ | ✘ |
| GQ252675/USA/1995 | II | ✓ | ✓ | ✘ | ✓ | ✓ | ✓ |
| GQ199885/USA/1996 | II | ✓ | ✘ | ✘ | ✓ | ✘ | ✘ |
| GQ199880/USA/1995 | II | ✓ | ✓ | ✘ | ✓ | ✓ | ✓ |
| GQ199879/USA/1994 | II | ✓ | ✓ | ✘ | ✓ | ✓ | ✓ |
| GQ199876/Venezuela/2007 | II | ✓ | ✓ | ✘ | ✓ | ✓ | ✓ |
| FJ882601/USA/1999 | II | ✓ | ✘ | ✘ | ✓ | ✘ | ✘ |
| FJ882598/USA/1998 | II | ✓ | ✓ | ✘ | ✓ | ✓ | ✓ |
| FJ882583/Venezuela/2007 | II | ✓ | ✘ | ✘ | ✓ | ✘ | ✘ |
| FJ850058/USA/1996 | II | ✓ | ✘ | ✘ | ✓ | ✘ | ✘ |
| FJ850057/USA/1995 | II | ✓ | ✓ | ✘ | ✓ | ✓ | ✓ |
| FJ639748/Venezuela/2000 | II | ✓ | ✓ | ✘ | ✓ | ✓ | ✓ |
| FJ639745/Venezuela/1999 | II | ✓ | ✓ | ✘ | ✓ | ✓ | ✓ |
| FJ639739/Venezuela/1998 | II | ✓ | ✓ | ✘ | ✓ | ✓ | ✓ |
| FJ226067/USA/1994 | II | ✓ | ✓ | ✘ | ✓ | ✓ | ✓ |
| FJ024476/Colombia/1997 | II | ✓ | ✓ | ✘ | ✓ | ✓ | ✓ |
| EU854295/USA/1986 | II | ✓ | ✘ | ✘ | ✓ | ✘ | ✘ |
| JQ822247/China/2010 | VI | ✓ | ✘ | ✘ | ✓ | ✘ | ✘ |
| JN983813/Brazil/2010 | II | ✓ | ✘ | ✘ | ✓ | ✘ | ✘ |
| JF262783/India/1961 | V | ✓ | ✓ | ✓ | ✓ | ✓ | ✓ |
| JF262782/Haiti/1994 | II | ✓ | ✓ | ✘ | ✓ | ✓ | ✓ |
| JF262781/Venezuela/1995 | II | ✓ | ✘ | ✘ | ✓ | ✘ | ✘ |
| JF262780/Malaysia/1973 | IV | ✓ | ✓ | ✘ | ✓ | ✓ | ✓ |
| JF262779/Malaysia/1975 | IV | ✓ | ✓ | ✘ | ✓ | ✓ | ✓ |
| FJ196850/China/1990 | I | ✓ | ✓ | ✘ | ✓ | ✓ | ✓ |
| GQ398256/Singapore/2005 | VI | ✓ | ✓ | ✘ | ✓ | ✓ | ✓ |
| GU289913/Colombia/1982 | II | ✓ | ✘ | ✘ | ✓ | ✘ | ✘ |
| GU318318/Puerto Rico/1985 | II | ✓ | ✘ | ✘ | ✓ | ✘ | ✘ |
| GU318310/Puerto Rico/1990 | II | ✓ | ✘ | ✘ | ✓ | ✘ | ✘ |
| GU318309/Puerto Rico/1991 | II | ✓ | ✘ | ✘ | ✓ | ✘ | ✘ |
| AY550909/Sri Lanka/1978 | I | ✓ | ✓ | ✘ | ✓ | ✓ | ✓ |
| AY618993/Thailand/2000 | VI | ✓ | ✓ | ✘ | ✓ | ✓ | ✓ |
| AY618992/Thailand/2001 | I | ✓ | ✓ | ✘ | ✓ | ✓ | ✓ |
| AY618991/Thailand/1977 | I | ✓ | ✓ | ✘ | ✓ | ✓ | ✓ |
| AY618990/Thailand/1991 | I | ✓ | ✓ | ✘ | ✓ | ✓ | ✓ |
| AY618989/Thailand/1997 | III | ✓ | ✓ | ✘ | ✓ | ✓ | ✓ |
| AF326573/Dominica/1981 | II | ✓ | ✘ | ✘ | ✓ | ✘ | ✘ |
| EF457906/Malaysia/1975 | IV | ✓ | ✓ | ✘ | ✓ | ✓ | ✓ |
| AY618988/Thailand/1997 | III | ✓ | ✓ | ✘ | ✓ | ✓ | ✓ |
| MN449006/Thailand/2012 | I | ✓ | ✓ | ✘ | ✓ | ✓ | ✓ |
| MN449004/Thailand/2012 | I | ✓ | ✘ | ✘ | ✓ | ✘ | ✘ |

**Note:** The highlighted sequences represent the study strains identified in our investigation during 2017 and 2018. The sequences are displayed in the phylogenetic tree and maximum clade credibility (mcc) tree with their original strain name given in the Table S2. The symbols ✓and ✘ denotes the sequences is used and not used respectively.
