# Supplementary material for: Global and local evolutionary dynamics of Dengue virus serotypes 1, 3, and 4
Source: Epidemiol Infect. 2023 Jun 9;151:e127. doi: 10.1017/S0950268823000924 (PMC10540175; doi:10.1017/S0950268823000924)
Supplement: Islam et al. supplementary material [file S0950268823000924sup002.pdf]

Global and Local Evolutionary Dynamics of Dengue Virus Serotype 1, 3 and 4.

Arshi Islam [1], Farah Deeba [1], Bansidhar Tarai [2], Ekta Gupta [3], Irshad H Naqvi [4], Mohd. Abdullah [4], Ravins Dohare [1], Anwar Ahmed [5], Fahad N. Almajhdi [5,6], Tajamul Hussain [5], Shama Parveen [1*]

[1] Centre for Interdisciplinary Research in Basic Sciences, Jamia Millia Islamia, New Delhi, India

[2] Department of Microbiology and Infection Control, Max Superspeciality Hospital, New Delhi, India

[3] Department of Clinical Virology, Institute of Liver and Biliary Sciences, New Delhi, India

[4] Dr. M.A. Ansari Health Centre, Jamia Millia Islamia, New Delhi, India

[5] Centre of Excellence in Biotechnology Research, College of Science, King Saud University, Riyadh, Saudi Arabia

[6] Department of Botany & Microbiology, College of Science, King Saud University, Riyadh, Saudi Arabia

*Corresponding author. Centre for Interdisciplinary Research in Basic Sciences, Jamia Millia Islamia, New Delhi, India

E-mail: sparveen2@jmi.ac.in

Figure S1

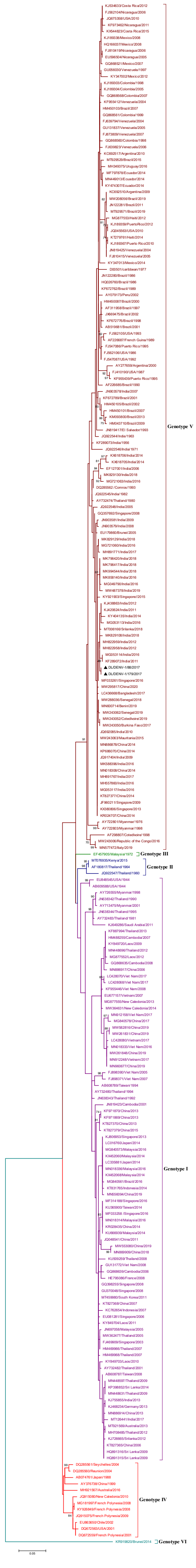

Genotype V
Genotype III
Genotype II
Genotype I
Genotype IV
Genotype VI

Figure S2

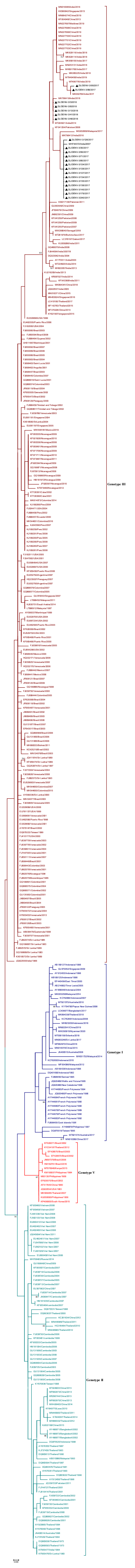

Figure S3

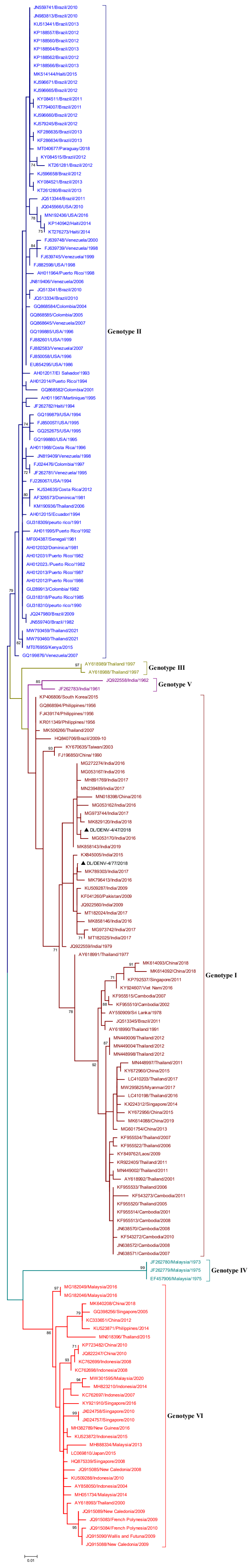

Genotype II
Genotype III
Genotype V
Genotype I
Genotype IV
Genotype VI
0.01

Figure S4A

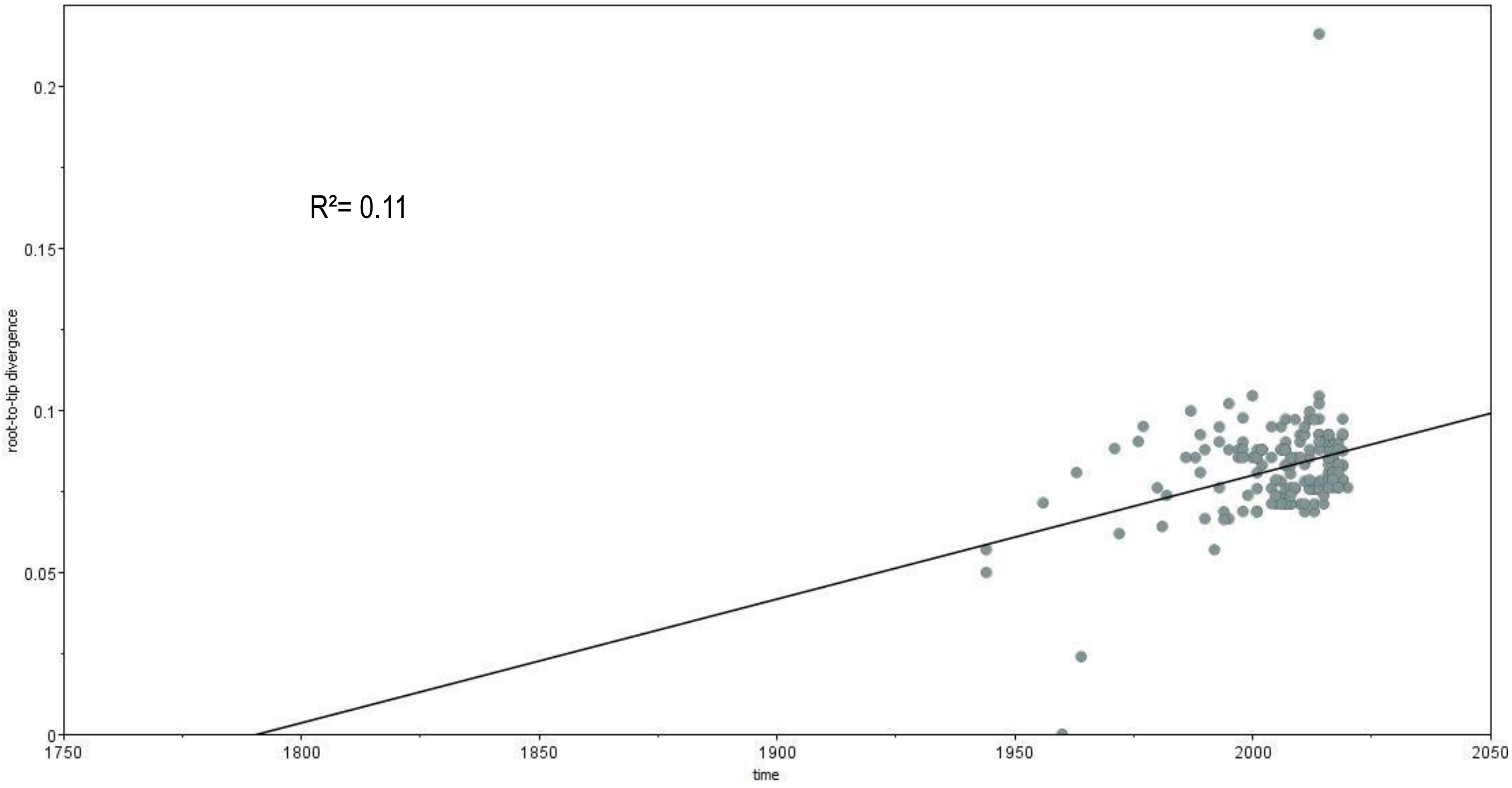

R²= 0.11
root-to-tip divergence
0.2
0.15
0.1
0.05
0
1750
1800
1850
1900
1950
2000
2050
time

Figure S4B

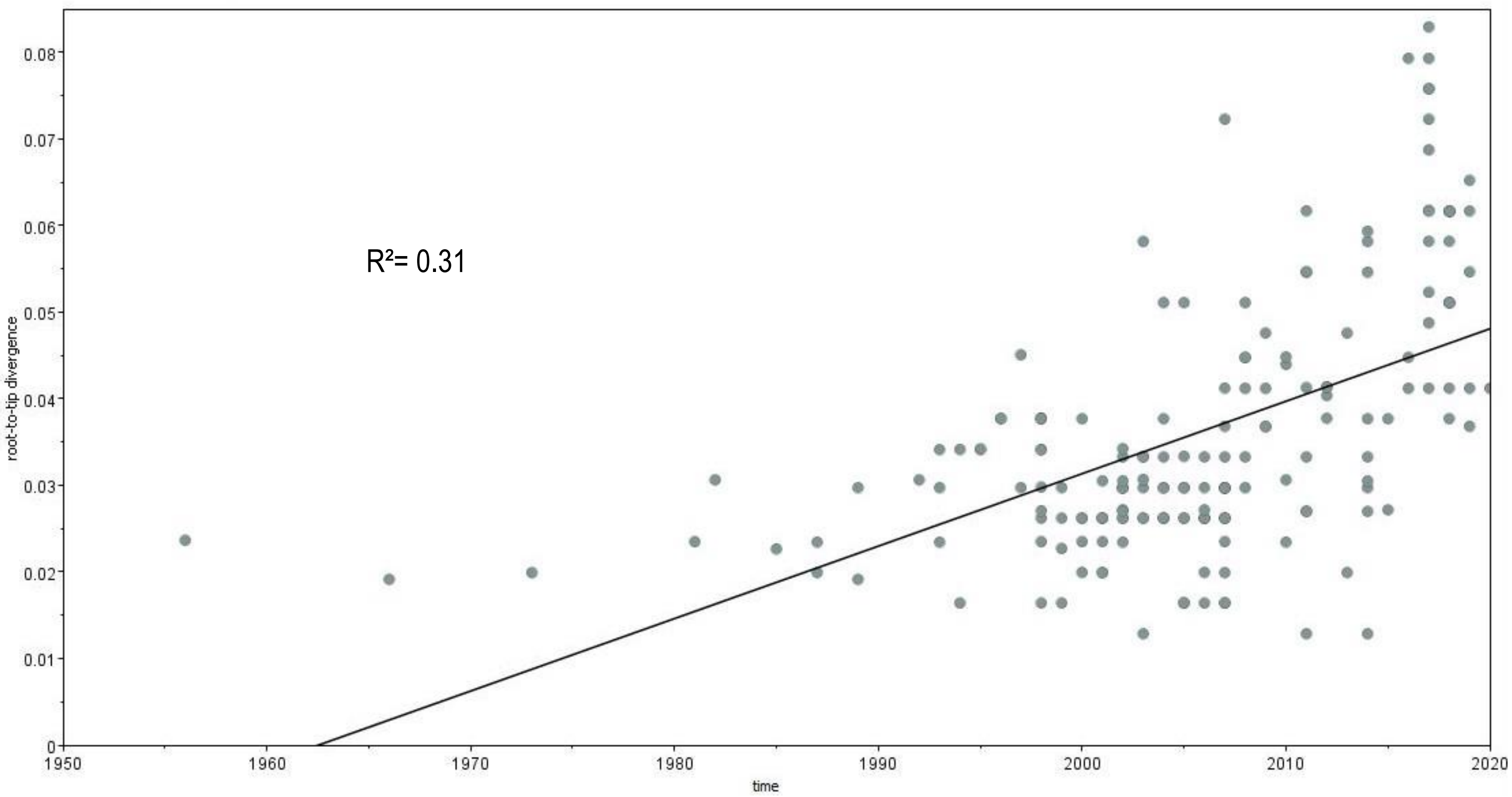

R^2= 0.31
0.08
0.07
0.06
0.05
0.04
0.03
0.02
0.01
0
root-to-tip divergence
1950
1960
1970
1980
1990
2000
2010
2020
time

Figure S4C

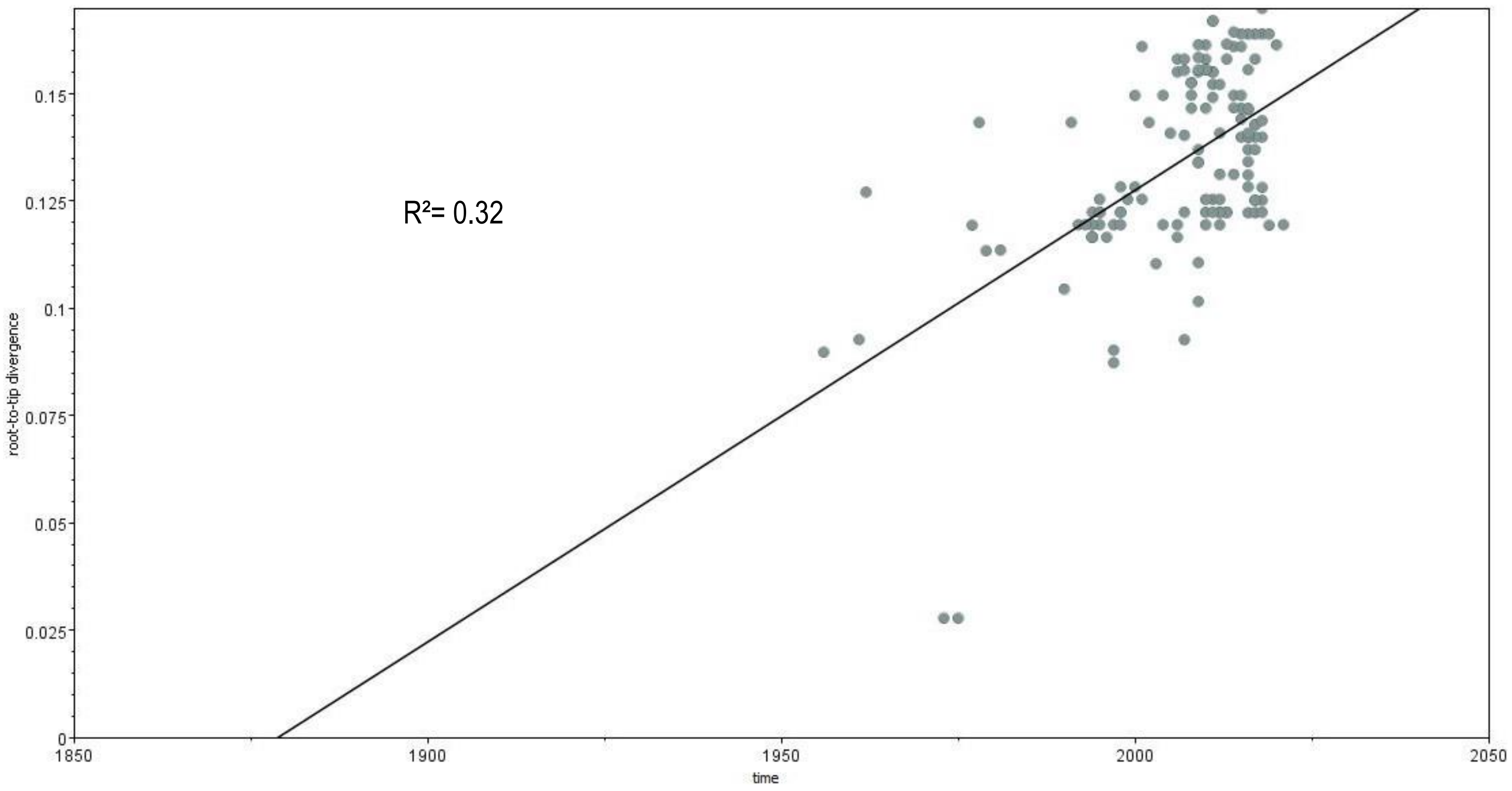
R²= 0.32
root-to-tip divergence
0
0.025
0.05
0.075
0.1
0.125
0.15
1850
1900
1950
2000
2050
time

Figure S5

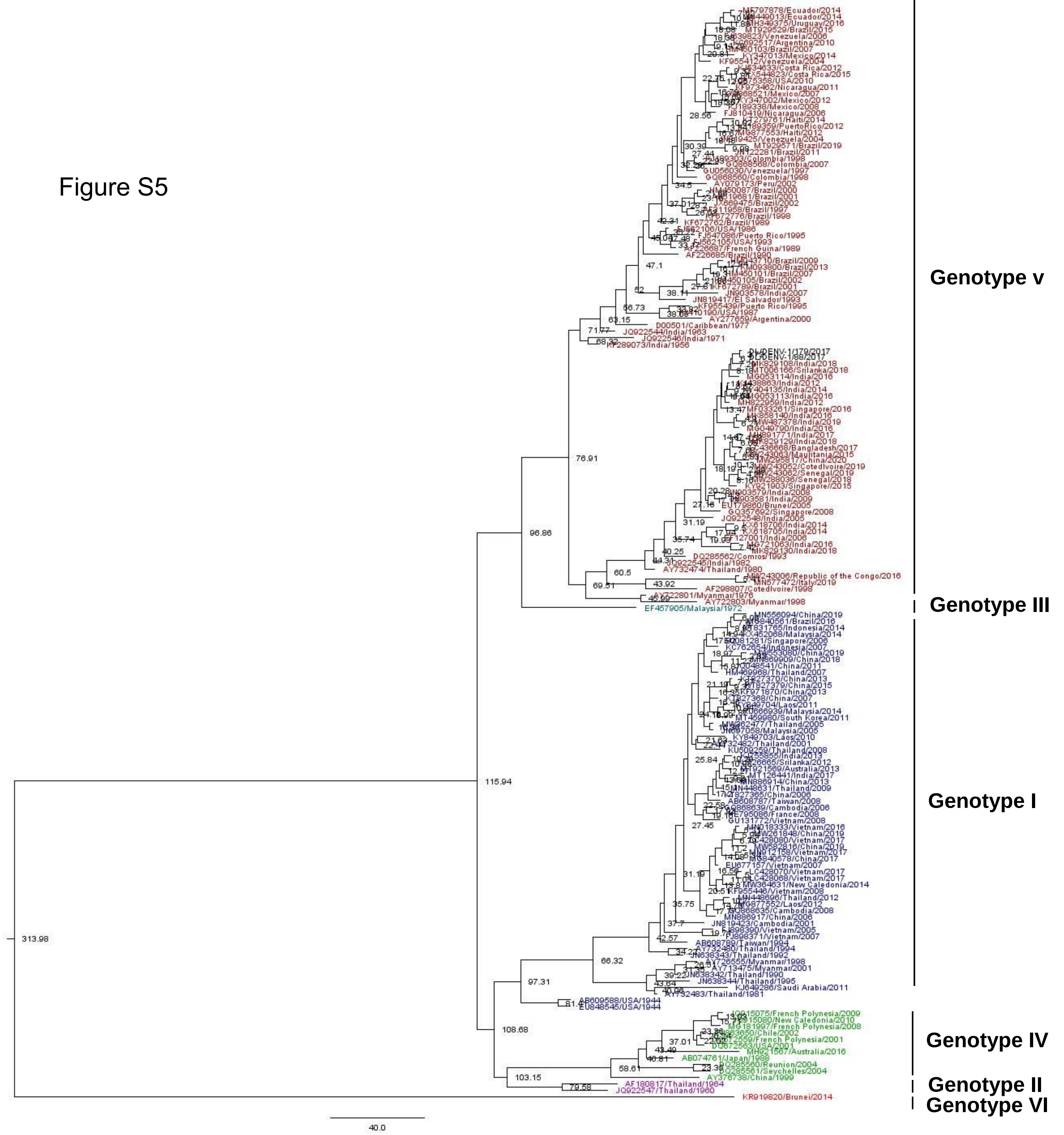
Genotype v
Genotype III
Genotype I
Genotype IV
Genotype II
Genotype VI
40.0

Figure S6

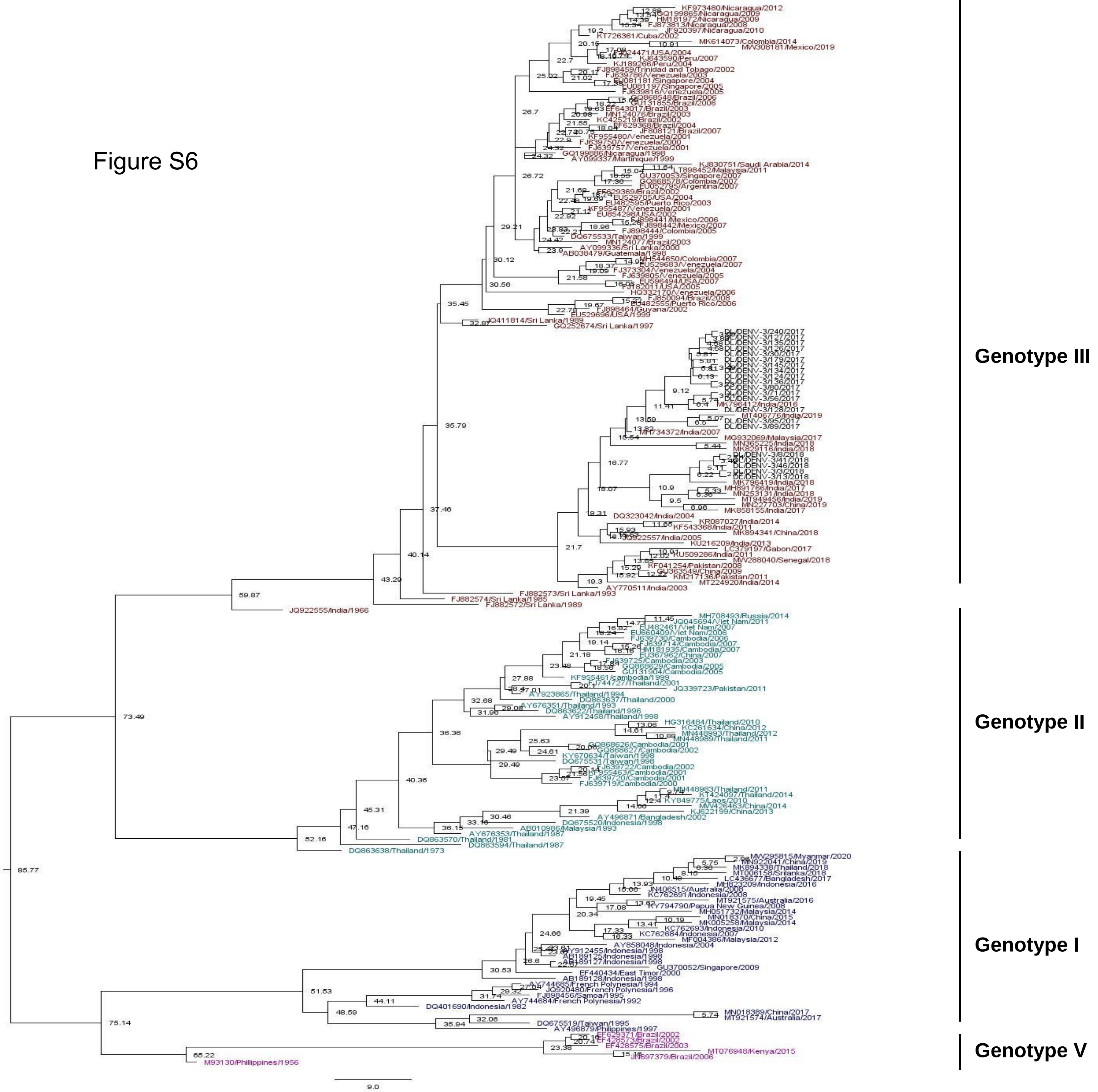

Figure S7

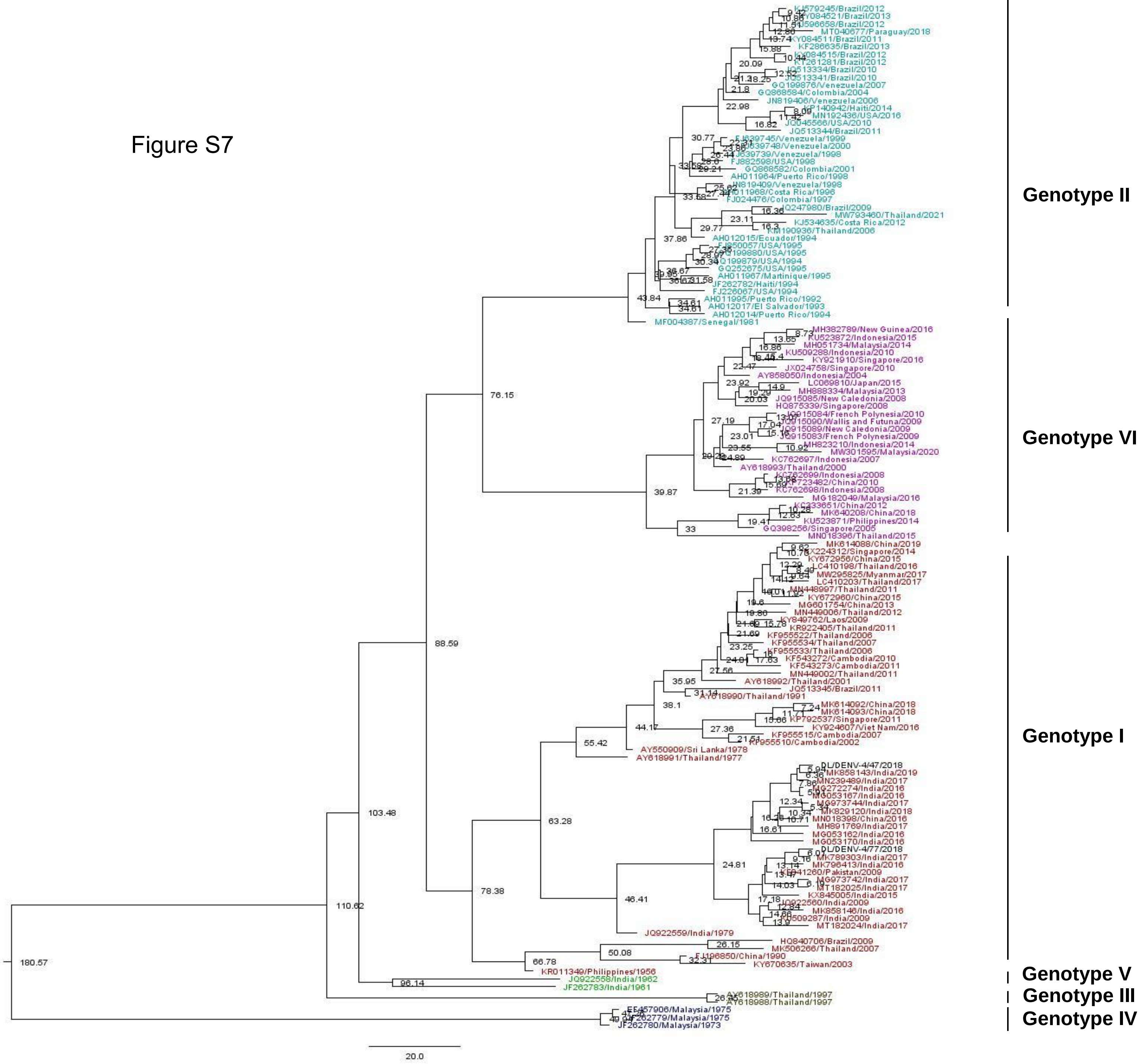
Genotype II
Genotype VI
Genotype I
Genotype V
Genotype III
Genotype IV
20.0
